# Food Insecurity in Peru: Measurement Properties of the FIES and Prevalence Estimates from the 2025 Household Survey

**DOI:** 10.3390/nu18152528

**Published:** 2026-08-04

**Authors:** Akram Hernández-Vásquez, Rodrigo Vargas-Fernández, Jamee Guerra Valencia

**Affiliations:** 1 Centro de Excelencia en Investigaciones Económicas y Sociales en Salud, Vicerrectorado de Investigación, Universidad San Ignacio de Loyola, Lima 15024, Peru; ahernandez@usil.edu.pe; 2 Epidemiology and Health Economics Research (EHER), Universidad Científica del Sur, Lima 15067, Peru; 3 Facultad de Ciencias de la Salud, Universidad Privada del Norte, Lima 15314, Peru; jamee.guerra@upn.pe

**Keywords:** food insecurity, food security, poverty, prevalence, public health, cross-sectional studies, surveys and questionnaires, Peru

## Abstract

**Background/Objectives**: Food insecurity (FI) is a major public health challenge and is monitored globally using the Food Insecurity Experience Scale (FIES). In Peru, the recent incorporation of the FIES into the National Household Survey (ENAHO) enables the generation of nationally representative estimates. This study evaluated the statistical validity of the FIES and estimated the prevalence of food insecurity among Peruvian households. **Methods**: A cross-sectional study was conducted using ENAHO 2025 data. FI was assessed using the eight-item FIES. Rasch analysis evaluated item fit, severity ordering, reliability, residual correlations, and differential item functioning. National prevalence estimates of moderate-or-severe and severe FI were calculated using the Food and Agriculture Organization of the United Nations (FAO) FIES methodology and household sampling weights. **Results**: The study included 32,041 households, representing approximately 9.9 million households nationwide. The FIES demonstrated satisfactory statistical validity, with a Rasch reliability of 0.796. Item severities followed the expected progression of FI, residual correlations remained below the recommended threshold, and no substantial differential item functioning was observed. The prevalence of moderate-or-severe FI was 30.9%, while severe FI affected 4.0% of households. Higher prevalence was observed among households living in extreme poverty, with unmet basic needs, lower educational attainment, permanent functional limitations, and in rural and Amazonian areas. **Conclusions**: The FIES demonstrated adequate validity for household-level monitoring of FI in Peru. Nearly one-third of households experienced moderate-or-severe FI, and prevalence varied across socioeconomic and geographic groups. The integration of the FIES into ENAHO provides a valuable platform for routine monitoring and food security policymaking.

## 1. Introduction

The concept of food security has evolved considerably over recent decades toward multidimensional frameworks that encompass food availability, access, and utilization [1]. In 1996, the Food and Agriculture Organization of the United Nations (FAO) defined food security as a condition in which all people, at all times, have physical, social, and economic access to sufficient, safe, and nutritious food that meets their dietary needs and food preferences for an active and healthy life [2]. This definition remains the most widely accepted internationally [1]. Food insecurity remains one of the major challenges for public health and sustainable development worldwide. In 2024, approximately 28% of the global population, representing more than two billion people, experienced moderate or severe food insecurity [3], with consequences extending beyond nutrition to affect physical health and overall well-being [4]. In this context, achieving food security and improved nutrition constitutes a central target of Sustainable Development Goal 2 (SDG 2) within the 2030 Agenda.

Although the prevalence of food insecurity has recently declined in Latin America and the Caribbean (LAC) [3], substantial social and territorial inequalities remain associated with this condition. Such disparities are also evident in the higher prevalence of food insecurity observed in rural areas and among women compared with urban areas and men [5,6,7,8,9,10,11]. Within this regional context, Peru is among the Latin American countries with the highest prevalence of moderate or severe food insecurity, with more than half of its population affected during the 2021–2023 period [12,13]. This represents a growing and complex public health issue that reflects persistent social, economic, and territorial inequalities in access to sufficient, safe, and nutritious food [14]. Such gaps may increase nutritional vulnerability, adversely affect population health, and hinder progress toward food security goals [4,15]. Despite the significant consequences of food insecurity, its monitoring remains limited in Peru due to the scarcity of frequent, regular, and methodologically rigorous measurements.

Monitoring food insecurity requires standardized instruments capable of generating estimates that are comparable across populations and over time [1]. In this regard, the Food Insecurity Experience Scale (FIES), developed by the FAO, has become one of the principal tools for measuring food insecurity [1]. The instrument assesses food insecurity through people’s experiences of difficulties in accessing food and is applicable at both the individual and household levels [1]. The FIES consists of eight items that progressively capture increasing levels of food insecurity severity, and its estimates are derived using the Rasch model within the framework of Item Response Theory, thereby enhancing the international comparability of results [16,17,18,19]. Furthermore, the FIES serves as the methodological basis for Sustainable Development Goal indicator 2.1.2, which monitors the prevalence of moderate or severe food insecurity in the population [18]. However, the statistical validity of the FIES has not been adequately established in Peru [20], which may compromise the reliability and comparability of prevalence estimates.

In Peru, the National Institute of Statistics and Informatics incorporated the FIES into the 2025 National Household Survey (Encuesta Nacional de Hogares, ENAHO) [21], providing an opportunity to generate updated, nationally representative estimates of food insecurity. However, without formal validation in the Peruvian context, the accuracy, validity, and comparability of these estimates remain uncertain. Given the complexity of measuring food insecurity, which is typically based on self-reported experiences and perceptions, and the lack of updated prevalence estimates to assess progress toward Sustainable Development Goal 2, this study aimed to statistically validate the Food Insecurity Experience Scale implemented in ENAHO 2025 and to estimate the prevalence of food insecurity among Peruvian households.

## 2. Materials and Methods

### 2.1. Study Design and Population

An analytical cross-sectional study was conducted using data from the 2025 ENAHO, executed by the National Institute of Statistics and Informatics (INEI) of Peru [22]. ENAHO is a probabilistic, stratified, multi-stage, and independent survey in each department, which allows for representative estimates at the national level, by area of residence (urban and rural), and by geographic regions [22]. The unit of analysis for this study was the household.

The following ENAHO modules were used: “Características de la Vivienda y del Hogar” (Housing and Household Characteristics), “Características de los Miembros del Hogar” (Household Member Characteristics), “Educación” (Education), “Salud” (Health), “Variables Calculadas (Sumarias)” (Summary) and “Inseguridad Alimentaria” (Food Insecurity). This last module incorporates the eight-item FIES [22], developed by the FAO [16,18], which is part of indicator 2.1.2 of the Sustainable Development Goals.

### 2.2. Population and Sample

The study population included all households surveyed in the 2025 ENAHO that had complete information in the module “Inseguridad Alimentaria” (Food Insecurity). The expansion factors provided by the INEI were applied to ensure the national representativeness of the estimates, considering the complex sample design of the survey. Of the 33,702 households with data recorded in the Food Insecurity module, 1637 (4.9%) had at least one unanswered item (Don’t know/No response) and were excluded. An additional 24 households were excluded because of missing data in other variables included in the analysis, resulting in a final analytical sample of 32,041 households.

### 2.3. Variables and Measurements

The main study variable was household-level food insecurity, measured using the FIES [18]. This scale consists of eight questions that assess food insecurity experiences during the month prior to the survey, covering dimensions of concern about food availability, diet quality and variety, reduction in quantities consumed, and experiences of hunger. Response options were dichotomous (Yes/No), with additional categories for “Don’t know” or “No response” cases. Based on the responses, households were classified into three categories: food security, moderate food insecurity, and severe food insecurity, using thresholds established by the FAO global FIES methodology [18]. The Spanish-language wording of the FIES items used in ENAHO 2025 reflects a national adaptation developed by INEI between 2023 and 2024, with technical support from FAO, including cognitive testing and a national pilot test, jointly validated by INEI and FAO prior to the module’s permanent incorporation into ENAHO in January 2025 [23]. The original Spanish wording of each item and its English translation are presented in Table 1.

Household variables used for descriptive analyses included demographic characteristics of the head of the household, such as sex, age (15–29, 30–59, 60–74, 75 or older), educational attainment (no formal education or preschool, primary, secondary, higher education), permanent functional limitation (yes/no), household size (1 member, 2–5 members, 6 or more members), household unmet basic needs (yes/no), and poverty level (extreme poor, poor, non-poor). Poverty level is a pre-calculated variable provided directly in the ENAHO 2025 database, based on comparing household per capita expenditure against the extreme poverty line (cost of a basic food basket) and the total poverty line (which additionally includes essential non-food goods and services). Households were classified as extreme poor, poor, or non-poor accordingly [24]. Additionally, geographic variables were considered: natural region (Coast, Highlands, Jungle), area of residence (urban, rural), geographic domain (Metropolitan Lima, urban Coast, rural Coast, urban Highlands, rural Highlands, urban Jungle, rural Jungle), and department of residence (25 departments of Peru, including the Constitutional Province of Callao). Permanent functional limitation of the household head was assessed through six questions on permanent limitations related to mobility, vision, communication, hearing, understanding or learning, and social interaction. The six disability questions administered by INEI as part of the Health module of ENAHO are based on the functional approach of the Washington Group Short Set (WG-SS) but constitute a nationally adapted instrument rather than a direct implementation. While ENAHO covers five of the six WG-SS domains (vision, hearing, mobility, communication, and cognition), it omits the self-care domain and includes a psychosocial functioning domain (relating to others) not present in the WG-SS framework [25]. Additionally, ENAHO employs binary yes/no responses rather than the WG-SS severity scale (no difficulty/some/a lot/cannot do at all). The household head’s responses were used as the household-level indicator of permanent functional limitation. Households were classified according to whether the head reported at least one permanent limitation among the six assessed items. Household unmet basic needs were assessed considering housing quality, overcrowding, sanitation, school attendance, and economic dependency. These five dimensions correspond to the official Unmet Basic Needs (Necesidades Básicas Insatisfechas, NBI) methodology used by INEI in Peru, based on the framework developed by the Economic Commission for Latin America and the Caribbean (CEPAL) and the United Nations Development Programme (UNDP) [26]. Households were classified as having no unmet basic needs (no) or at least one unmet basic need (yes).

### 2.4. Statistical Analysis

Data management and analysis were performed using R software version 4.6.0 (R Foundation for Statistical Computing, Vienna, Austria) and the FIES web application developed by the FAO available at https://foodandagricultureorganization.shinyapps.io/fies/ (accessed on 2 June 2026) [27]. This application allows for the analysis of FIES data through the application of the Rasch model, an Item Response Theory (IRT) method that evaluates the validity and reliability of the scale and produces globally comparable prevalence estimates [27].

A descriptive analysis of household characteristics was performed according to food insecurity categories. Categorical variables were presented using weighted absolute and relative frequencies. Estimates were weighted using the survey expansion factors and accounted for the complex sampling design of the survey.

Statistical validation of the FIES data was performed using the FAO FIES application according to the procedures specified in the FIES methodology manual [27]. The application automatically evaluated Rasch model fit statistics (infit and outfit), Rasch reliability, and residual correlations between items. Respondents with extreme raw scores (0 or 8 affirmative responses) were excluded from the estimation of item parameters because they do not contribute information to the calibration process, although they were retained for the final prevalence estimation. The equating procedure was conducted by calibrating item severity parameters against the global FIES reference scale, allowing for international comparability of the estimates [18,27]. As an additional sensitivity analysis, respondents with extreme outfit statistics (≥99.74th percentile) were excluded and the Rasch model was re-estimated to evaluate the stability of model fit statistics.

Prevalence estimates of moderate-or-severe food insecurity (SDG indicator 2.1.2) and severe food insecurity were obtained using the probability-based assignment approach implemented in the FAO FIES methodology [27]. All estimates accounted for the complex survey design, and their precision was assessed using margins of error (MoE), obtained directly from the FAO FIES web application [27]. Corresponding 95% confidence intervals were calculated as the point estimate ± MoE.

Maps and graphical representations of the prevalence of moderate-or-severe and severe food insecurity across the departments of Peru were generated using Python version 3.14 (Python Software Foundation, Wilmington, DE, USA) and the GeoPandas, Matplotlib, Pandas, and NumPy packages.

### 2.5. Ethical Considerations

The study used publicly available secondary data from the ENAHO executed by the INEI. The survey guarantees the anonymity of the participants, and the databases are published without personal data that would allow for the identification of the participants.

## 3. Results

The study sample included 32,041 households, which represented 9,917,263 households after applying sample weights (Table 2). 61.5% corresponded to male heads of household, and 38.5% to female heads of household. The majority of household heads were between 30 and 59 years old (58.6%), lived in urban areas (78.7%), and were not in poverty (79.6%). By natural region, 52.9% of households were located on the Coast, 34.7% in the Highlands, and 12.3% in the Jungle.

The Rasch analysis showed a progressive ordering of FIES item severity. In the total sample, the items with the lowest severity were WORRIED (−3.04), HEALTHY (−2.14), and FEWFOOD (−1.83), while those with the highest severity were RUNOUT (1.61), HUNGRY (1.74), and WHLDAY (3.79) (Table 3). Rasch reliability was adequate, with a value of 0.796 in the total sample and 0.798 in the analytical subsample. Fit statistics showed infit values close to 1 for most items; however, outfit values were elevated for WORRIED and WHLDAY in the total sample.

The weighted distribution of the raw score showed that 41.2% of households reported no experiences of food insecurity, while 3.5% reached the maximum score of 8 (Table 4). Raw score severity parameters increased monotonically, from −4.139 for score 0 to 4.447 for score 8, supporting the expected ordinal structure of the scale.

The residual correlation matrix generally showed low values between item pairs, supporting the assumption of conditional independence of the Rasch model (Table 5). Most of the residual correlations were less than 0.20 in absolute value. The highest residual correlation was observed between the HEALTHY and FEWFOOD items (r = 0.38), followed by RUNOUT and HUNGRY (r = 0.24), while the rest of the correlations remained within ranges considered acceptable. No residual correlations > 0.40 were identified.

The estimated national prevalence of moderate or severe food insecurity was 30.9% (MoE ± 0.7), while severe food insecurity was 4.0% (MoE ± 0.3) (Table 6). Households with female heads of household had a higher prevalence of moderate or severe food insecurity than those with male heads of household (35.1% vs. 28.3%), as well as higher severe food insecurity (5.1% vs. 3.4%). Likewise, moderate or severe food insecurity was higher in households with heads with no education or preschool education (45.6%) and lower in households with heads with higher education (17.9%). It was also more frequent in households whose head had a permanent functional limitation (41.4%), households with unmet basic needs (41.9%), and households in extreme poverty (51.9%). Geographically, the prevalence of moderate or severe food insecurity was highest in the Jungle (34.3%). Prevalence was also higher in rural than urban areas (37.3% vs. 29.2%). By geographic domain, the highest prevalence was observed in the rural Jungle (38.7%).

**Figure 1:**
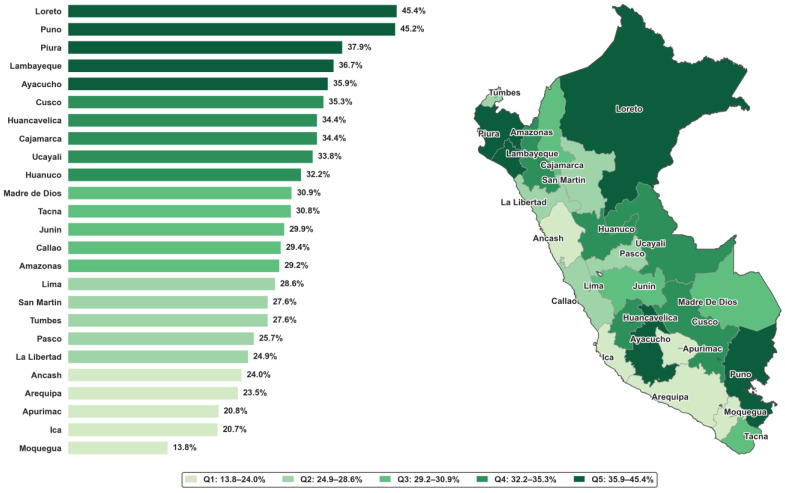
Department-level prevalence of moderate-or-severe food insecurity in Peru, 2025.

At the departmental level, the highest prevalences of moderate or severe food insecurity were observed in Loreto (45.4%), Puno (45.2%), Piura (37.9%), Lambayeque (36.7%), and Ayacucho (35.9%) (Figure 1). The lowest prevalences corresponded to Moquegua (13.8%), Ica (20.7%), Apurimac (20.8%), Arequipa (23.5%), and Ancash (24.0%).

**Figure 2:**
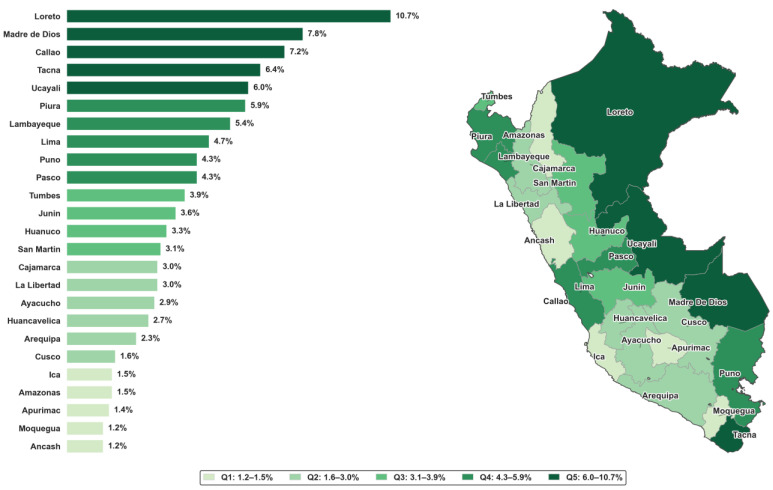
Department-level prevalence of severe food insecurity in Peru, 2025.

For severe food insecurity, the highest prevalences were observed in Loreto (10.7%), Madre de Dios (7.8%), Callao (7.2%), Tacna (6.4%), and Ucayali (6.0%) (Figure 2).

Differential item functioning was assessed across the population subgroups considered in the analysis, and no substantial differential item functioning was evident from the graphical assessment. The complete output generated by the FIES web application, including differential item functioning plots for all evaluated subgroups, is provided in the Supplementary Materials (Tables S1–S8, Figures S1–S26).

## 4. Discussion

This study conducted the statistical validation of the Food Insecurity Experience Scale and estimated the prevalence of food insecurity in Peru using nationally representative data from the ENAHO. Overall, the FIES demonstrated satisfactory psychometric performance, with acceptable reliability, item fit statistics, and conditional independence, supporting its validity for measuring food insecurity in the Peruvian population. Based on calibrated FIES estimates, the national prevalence of moderate or severe food insecurity reached 30.9%, while severe food insecurity affected 4.0% of households. Because food insecurity was assessed at the household level, all sociodemographic characteristics examined (sex, age, education, and permanent functional limitation) refer to the household head and serve as proxies for household-level conditions rather than individual-level attributes. In addition, food insecurity was unevenly distributed, with substantially higher prevalence observed among households living in poverty, those with unmet basic needs, household whose heads had lower educational attainment, and households whose head reported permanent functional limitations. Marked geographic variation was also identified, with the highest prevalence concentrated in rural and Amazonian areas and in departments such as Loreto and Puno.

The statistical validation of the FIES indicated that the scale met the main criteria recommended for the assessment of food insecurity data. Reliability values were 0.796 in the full sample and 0.798 in the analytical subsample, exceeding the minimum threshold of 0.70 recommended for eight-item FIES applications [18]. In addition, all items exhibited infit statistics within the recommended range of 0.7–1.3 (WORRIED AND WHLDAY showing the largest infit values), indicating consistency with the assumption of equal discrimination across the food insecurity severity continuum [16]. Residual correlations between item pairs remained below the threshold commonly used to indicate conditional dependence, supporting the assumption that the items measured a common underlying construct while retaining sufficient independence from one another [18]. Although elevated outfit values were observed for the WORRIED and WHLDAY items in the full sample, outfit statistics are particularly sensitive to infrequent and unexpected response patterns and are primarily intended to identify potential outliers rather than assess overall model fit [18]. These values remained below thresholds reported as acceptable in regional FIES applications (e.g., <5) [23], indicating limited practical impact on the interpretation of the scale. Importantly, outfit values in the analytical subsample used for formal validation were within acceptable limits (i.e., <2), supporting the adequacy of the scale for population-level assessment of food insecurity in Peru.

Overall, the psychometric performance of the FIES observed in Peru is consistent with findings reported in previous studies conducted in diverse settings. Similar results have been reported in a multi-country assessment involving countries from Latin America and the Caribbean [16] and in national applications conducted in Bangladesh, the Bahamas, Chile, and the United Kingdom [28,29,30,31]. Despite this overall consistency, minor variations in item severity ordering have been documented across settings. For example, Jubayer et al. reported a reversal in the ordering of the ATELESS and SKIPPED items [28], a pattern also observed in the present study. Because both items represent moderate levels of food insecurity severity, such variations are unlikely to affect prevalence estimation and may instead reflect differences in the sequence of coping strategies adopted by households while experiencing food insecurity. For example, while some households may first reduce portion sizes, others may begin by skipping meals, leading to minor variations in the relative ordering of adjacent items [32]. Together, these findings reinforce the suitability of the FIES for use in national surveys and population-level monitoring of food insecurity.

Using the validated FIES, we estimated that nearly one-third of the Peruvian population experienced moderate or severe food insecurity, while severe food insecurity affected 4.0% of the population. These findings indicate that food insecurity remains an important public health and social challenge in Peru, affecting a substantial proportion of the population [14], despite sustained economic growth and substantial poverty reduction over the past two decades [33]. The prevalence estimate observed in the present study was higher than that reported in an earlier FIES-based assessment for Peru. Using data from the Gallup World Poll, Smith et al. reported that approximately one-quarter of the Peruvian population experienced food insecurity during 2015 [34], compared with 30.9% observed in the present study. In contrast, the most recent edition of The State of Food Security and Nutrition in the World estimated that 41.0% of the Peruvian population experienced moderate or severe food insecurity and 14.5% experienced severe food insecurity during 2022–2024 [3], compared with the lower estimates observed in the present study. These differences should be interpreted in light of both temporal and methodological differences across the available estimates. While both the estimates reported by Smith et al. and those published in the State of Food Security and Nutrition reports were derived from FIES data collected through the Gallup World Poll, the present study was based on the first nationally representative implementation of the FIES within Peru’s official household survey system. This distinction is particularly relevant because the Gallup World Poll was originally intended to provide FIES-based estimates in countries where nationally representative FIES data were not yet available [3]. Furthermore, FAO acknowledges that differences in survey sources and sample sizes may contribute to variability in food insecurity estimates, particularly when data are generated through different monitoring systems [3]. In contrast, the incorporation for the first time of the FIES into ENAHO provides estimates derived from Peru’s official national household survey, which uses a substantially larger sample and enables disaggregation across geographic and socioeconomic subgroups. Moreover, FAO identifies nationally implemented surveys as the preferred source for monitoring Sustainable Development Goal indicator 2.1.2 whenever such data are available [3]. Therefore, beyond generating updated prevalence estimates, the integration of the FIES into ENAHO represents an important step toward institutionalizing the monitoring of food insecurity in Peru and strengthening the evidence base available for food security policies and programs.

In the present study, food insecurity was markedly concentrated among socially and economically disadvantaged groups. While approximately one-third of the Peruvian households experienced moderate or severe food insecurity, the prevalence increased to nearly one-half among households whose head had no formal or preschool education (45.6%) and exceeded 50% among those households living in extreme poverty. A clear social gradient was also observed across poverty categories, educational attainment, and unmet basic needs, with food insecurity becoming progressively less frequent as socioeconomic conditions improved. For example, the prevalence of moderate or severe food insecurity declined from 51.9% among households living in extreme poverty to 27.5% among those classified as non-poor, and from 45.6% among households whose head had no formal or preschool education to 17.9% among those with higher education. These findings are consistent with previous evidence from Latin America and other developing regions showing that food insecurity disproportionately affects populations with fewer economic and educational resources [34,35,36]. Food insecurity was also more frequent among female-headed households, a pattern previously documented in studies from Latin America and other settings [34,36]. Similarly, individuals reporting permanent functional limitations exhibited a higher prevalence of food insecurity (41.4% vs. 30.0%). Previous studies have consistently identified disability as a marker of increased vulnerability to food insecurity, potentially reflecting a combination of reduced income-generating opportunities, higher health-related expenditures, and barriers to food access and acquisition [37]. Together, these patterns indicate that the burden of food insecurity in Peru is concentrated among groups facing greater social and material disadvantage.

Marked geographic disparities in food insecurity were observed across Peru’s administrative departments. Higher prevalence of moderate or severe food insecurity was identified in rural areas, the Amazon region, and departments such as Loreto and Puno, where nearly one-half of households experienced some degree of food insecurity. These findings are consistent with the persistence of territorial inequalities in living conditions, infrastructure, and access to services across the country, particularly in geographically remote areas [33]. These disparities may partly reflect greater geographic isolation in Amazonian departments such as Loreto, where communities often depend on costly river transportation and face substantial travel times to access services [38], and where the transport of agricultural products also faces significantly higher costs than road-based systems [39], as well as a stronger reliance on subsistence agriculture and vulnerability to climatic shocks in Andean highland departments, both of which can constrain stable access to food. However, the geographic distribution of severe food insecurity was not entirely aligned with that of moderate or severe food insecurity. While Loreto exhibited both the highest overall food insecurity and the highest prevalence of severe food insecurity, departments such as Callao, Madre de Dios, and Tacna also showed relatively high levels of severe food insecurity despite not ranking among those with the highest overall prevalence. Conversely, Puno presented one of the highest prevalence of moderate or severe food insecurity but a comparatively lower prevalence of severe food insecurity. These findings suggest that the burden and intensity of food insecurity may vary across territories and highlight the importance of examining both moderate-or-severe and severe food insecurity when identifying priority populations and geographic areas for intervention.

As the first nationally representative assessment of food insecurity derived from the implementation of the FIES within Peru’s official household survey system, our findings have important implications for food security monitoring and policy development in Peru. Although nearly one-third of the households experienced moderate or severe food insecurity, substantial differences were observed across socioeconomic groups and administrative departments. These patterns suggest that national estimates alone may mask important inequalities and that interventions aimed at improving food security should consider both social and territorial vulnerabilities. The incorporation of the FIES into ENAHO provides a valuable opportunity to routinely monitor food insecurity using a nationally representative platform, identify priority populations and geographic areas, and evaluate progress over time. These patterns, such as the higher burden among households in extreme poverty or with lower educational attainment, and the concentration of cases in departments like Loreto and Puno, could inform the geographic and population-based targeting of food assistance and social protection programs toward those most affected. The marked territorial disparities observed in this study also underscore the multidimensional nature of food security. Because the FIES captures experiences related to food access, the observed inequalities suggest that food insecurity cannot be understood solely in terms of food availability. Rather, economic, social, and territorial conditions may play an important role in shaping access to adequate food across populations. In this context, reducing food insecurity will likely require coordinated actions across food, health, and social protection systems that address the factors influencing access to food among vulnerable populations. Beyond providing updated prevalence estimates, the routine implementation of the FIES within ENAHO offers an opportunity to move from episodic assessments of food insecurity toward a sustained monitoring system capable of identifying emerging vulnerabilities, tracking inequalities, and informing evidence-based responses across sectors. Such a system may also strengthen the evaluation of food and social protection policies and enhance accountability toward national and global food security commitments, including Sustainable Development Goal 2.

This study has some limitations. First, although the FIES is a validated instrument for monitoring food insecurity, it primarily captures experiences related to food access and does not directly assess other dimensions of food security, such as food availability, utilization, or stability. Therefore, the findings should be interpreted as reflecting inequalities in access to food rather than the full spectrum of food security. Second, the eight FIES items were self-reported and referred to experiences during the previous month. Shorter recall periods are recommended by FAO because they reduce recall error compared with the standard 12-month period [40]. As such, responses may be affected by recall errors and social desirability bias. Third, the one-month recall period may also be influenced by seasonal variation [41]. Given the marked climatic, agricultural, and socioeconomic heterogeneity across Peruvian regions, food insecurity experiences may vary according to the timing and location of data collection, potentially introducing unmeasured seasonal effects. Fourth, the exclusion of households with incomplete information in the food insecurity module may have introduced selection bias if non-response was not random. This possibility is particularly relevant because the FIES was only recently incorporated into ENAHO, a multipurpose survey that was not originally designed specifically to measure food insecurity. Fifth, the analyses were based on data collected during a single survey year, precluding the assessment of temporal trends or changes in food insecurity over time. Future analyses using repeated rounds of ENAHO will help evaluate the evolution of food insecurity and the persistence of the socioeconomic and territorial disparities identified in this study. Sixth, food insecurity was measured at the household level. Consequently, the estimates do not reflect the experiences of individual household members. In addition, some characteristics used for disaggregation, including sex, age, educational attainment, and functional limitation, corresponded to the head of household rather than to all household members. Therefore, these results should be interpreted as differences between households according to the characteristics of their head rather than as estimates for individual population groups. Seventh, because this represents the first implementation of the FIES within ENAHO, no previous nationally representative estimates based on the same instrument and sampling framework are available. This limits direct comparisons with historical estimates derived from the Gallup World Poll, which relies on different sampling and survey procedures. Finally, although no residual correlation exceeded the threshold of 0.40 recommended for FIES validation, the correlation between HEALTHY and FEWFOOD (r = 0.38) was the highest observed in the scale. This may reflect the close conceptual relationship between reductions in dietary quality and dietary diversity, which are commonly observed during the earlier stages of food insecurity before more severe quantitative restrictions occur [42].

## 5. Conclusions

In conclusion, the FIES demonstrated satisfactory statistical validity when applied within Peru’s national household survey system, supporting its use for population-level monitoring of food insecurity. Approximately one in every three households in Peru experienced moderate or severe food insecurity according to ENAHO 2025, underscoring its persistence as a major public health challenge. The marked socioeconomic and territorial inequalities identified in this study indicate that the burden of food insecurity disproportionately affects socially and economically vulnerable groups. The incorporation of the FIES into ENAHO provides a valuable opportunity to strengthen routine monitoring and inform policies aimed at reducing inequalities in access to adequate food.

## Figures and Tables

**Table 1:** Food Insecurity Experience Scale (FIES) items: original Spanish wording and English translation.

Item	Original Spanish Wording	English Translation
1	En el mes anterior, ¿hubo un momento en el que usted o algún otro miembro del hogar estuvo preocupado por no tener suficiente comida para comer por falta de dinero u otras formas de conseguir los alimentos?	In the past month, was there a time when you or someone else in your household worried about not having enough food to eat because of a lack of money or other resources to obtain food?
2	Pensando aún en el mes anterior, ¿hubo un momento en el que usted o algún otro miembro del hogar no pudo comer alimentos saludables y nutritivos debido a la falta de dinero u otras formas de conseguir los alimentos?	Still thinking about the past month, was there a time when you or someone else in your household were unable to eat healthy and nutritious food because of a lack of money or other resources to obtain food?
3	En el mes anterior, ¿hubo un momento en el que usted o algún otro miembro del hogar no comió alimentos variados debido a la falta de dinero u otras formas de conseguir los alimentos?	Was there a time when you or someone else in your household ate only a few kinds of foods because of a lack of money or other resources to obtain food?
4	En el mes anterior, ¿hubo un momento en el que usted o algún otro miembro del hogar tuvo que dejar de desayunar, almorzar o cenar porque no había suficiente dinero u otras formas de conseguir los alimentos?	Was there a time when you or someone else in your household had to skip a meal (breakfast, lunch, or dinner) because there was not enough money or other resources to obtain food?
5	Pensando aún en el mes anterior, ¿hubo un momento en el que usted o algún otro miembro del hogar comió menor cantidad de lo normal, debido a la falta de dinero u otras formas de conseguir los alimentos?	Still thinking about the past month, was there a time when you or someone else in your household ate less than you thought you should because of a lack of money or other resources to obtain food?
6	En el mes anterior, ¿hubo un momento en el que su hogar se quedó sin alimentos por la falta de dinero u otras formas de conseguir los alimentos?	Was there a time when your household ran out of food because of a lack of money or other resources to obtain food?
7	En el mes anterior, ¿hubo un momento en el que usted o algún otro miembro del hogar tuvo hambre, pero no comió porque no había suficiente dinero u otras formas de conseguir los alimentos?	Was there a time when you or someone else in your household were hungry but did not eat because there was not enough money or other resources to obtain food?
8	En el mes anterior, ¿hubo un momento en el que usted o algún otro miembro del hogar estuvo sin comer durante un día entero debido a la falta de dinero u otras formas de conseguir los alimentos?	Was there a time when you or someone else in your household went without eating for a whole day because of a lack of money or other resources to obtain food?

Spanish wording corresponds to the household survey questionnaire. Response options/Opciones de respuesta: 1 = Sí/Yes, 2 = No/No, 3 = No sabe/Don’t know, 4 = No responde/No response.

**Table 2:** Characteristics of household heads and households included in the study, Peru, 2025.

Characteristic	*n*	Weighted *n*	Weighted %
Overall	32,041	9,917,263	100
Characteristics of household head			
Sex of household head			
Male	20,247	6,102,629	61.5
Female	11,794	3,814,634	38.5
Age of household head (years)			
15–29	1615	502,704	5.1
30–59	18,586	5,814,060	58.6
60–74	8242	2,554,062	25.8
≥75	3598	1,046,437	10.6
Educational attainment of household head			
No formal education or preschool	1883	545,232	5.5
Primary education	9933	2,819,527	28.4
Secondary education	12,304	4,139,457	41.7
Higher education	7921	2,413,047	24.3
Permanent functional limitation of household head			
No	29,464	9,149,112	92.3
Yes	2577	768,150	7.7
Household characteristics			
Household size (members)			
1 member	5588	1,565,086	15.8
2–5 members	23,523	7,418,778	74.8
≥6 members	2930	933,398	9.4
Household unmet basic needs			
No	26,577	8,517,608	85.9
Yes	5464	1,399,655	14.1
Household poverty status			
Extreme poor	1138	356,633	3.6
Poor	4711	1,662,431	16.8
Non-poor	26,192	7,898,198	79.6
Geographic characteristics			
Natural region			
Coast	13,424	5,247,543	52.9
Highlands	11,609	3,445,352	34.7
Jungle	7008	1,224,367	12.3
Area of residence			
Rural	11,290	2,115,969	21.3
Urban	20,751	7,801,294	78.7
Geographic domain			
Metropolitan Lima	3392	2,887,619	29.1
Urban Coast	8379	2,157,077	21.8
Rural Coast	1653	202,847	2.0
Urban Highlands	4963	1,934,971	19.5
Rural Highlands	6646	1,510,381	15.2
Urban Jungle	4017	821,627	8.3
Rural Jungle	2991	402,740	4.1
Department of residence			
Amazonas	1177	140,561	1.4
Ancash	1351	360,677	3.6
Apurimac	868	162,153	1.6
Arequipa	1465	419,360	4.2
Ayacucho	1136	264,821	2.7
Cajamarca	1413	519,403	5.2
Callao	881	297,128	3.0
Cusco	1160	463,937	4.7
Huancavelica	1017	177,275	1.8
Huanuco	1208	299,351	3.0
Ica	1482	264,181	2.7
Junin	1465	466,016	4.7
La Libertad	1508	610,507	6.2
Lambayeque	1383	395,069	4.0
Lima	3866	2,882,696	29.1
Loreto	1338	265,761	2.7
Madre de Dios	583	51,807	0.5
Moquegua	905	70,469	0.7
Pasco	830	105,283	1.1
Piura	1591	546,075	5.5
Puno	1037	536,978	5.4
San Martin	1317	278,918	2.8
Tacna	1144	120,138	1.2
Tumbes	830	78,540	0.8
Ucayali	1086	140,159	1.4

*n* is the unweighted number of households. Weighted *n* corresponds to the estimated number of households in the Peruvian population. Percentages were calculated using household sampling weights and account for the complex survey design of ENAHO 2025. Household head refers to the person identified as the head of the household in the survey. Percentages may not sum to 100 due to rounding. Household poverty status was classified as extreme poor, poor, and non-poor according to the official poverty classification used by the National Institute of Statistics and Informatics (INEI).

**Table 3:** Comparison of Rasch item parameters and fit statistics between the full sample and the subsample, Peru, 2025.

	Full Sample					Subsample			
Item	Severity	SE	Infit	Outfit		Severity	SE	Infit	Outfit
WORRIED	−3.042	0.024	1.211	3.369		−3.083	0.025	1.210	2.130
HEALTHY	−2.136	0.022	0.792	1.632		−2.162	0.022	0.797	1.723
FEWFOOD	−1.826	0.021	0.858	1.283		−1.849	0.021	0.863	1.338
ATELESS	−0.680	0.021	0.931	1.026		−0.696	0.021	0.937	1.043
SKIPPED	0.706	0.024	0.913	0.972		0.698	0.024	0.922	1.000
RUNOUT	1.613	0.028	0.945	1.360		1.614	0.028	0.958	1.399
HUNGRY	1.742	0.028	0.866	1.210		1.745	0.029	0.877	1.236
WHLDAY	3.785	0.047	1.037	3.650		3.890	0.049	1.023	1.095

SE: standard error. Severity parameters and fit statistics were estimated using the Rasch model. Acceptable fit is generally indicated by Infit values between 0.7 and 1.3. The subsample excluded respondents with extreme misfit according to the FIES methodological guidelines. Rasch reliability: full sample = 0.796; calibrated subsample = 0.798.

**Table 4:** Raw score statistics from the Food Insecurity Experience Scale (FIES) Rasch model, Peru, 2025.

Raw Score	Severity Parameter	Standard Error (SE)	Weighted Number	Weighted Proportion (%)
0	−4.139	1.547	4,090,822	41.2
1	−3.280	1.212	1,052,183	10.6
2	−2.062	1.021	862,402	8.7
3	−1.032	1.000	1,252,555	12.6
4	−0.044	1.006	882,263	8.9
5	0.960	1.005	643,085	6.5
6	2.028	1.068	398,356	4.0
7	3.428	1.314	384,984	3.9
8	4.447	1.547	350,614	3.5

Severity parameters and standard errors were derived from the Rasch model calibration. The extreme raw scores (0 and 8) have extrapolated severity values.

**Table 5:** Residual correlation matrix of the Food Insecurity Experience Scale (FIES) items, Peru, 2025.

Item	WORRIED	HEALTHY	FEWFOOD	SKIPPED	ATELESS	RUNOUT	HUNGRY	WHLDAY
WORRIED	1	0.12	−0.09	−0.10	−0.17	−0.11	−0.12	−0.12
HEALTHY		1	0.38	−0.02	−0.07	−0.09	−0.10	−0.10
FEWFOOD			1	0.01	0.02	−0.08	−0.09	−0.10
SKIPPED				1	0.17	0.02	0.06	−0.06
ATELESS					1	0.06	0.06	−0.04
RUNOUT						1	0.24	0.06
HUNGRY							1	0.20
WHLDAY								1

Residual correlations greater than 0.40 are commonly considered indicative of potential local dependence between item pairs. No residual correlation exceeded this threshold.

**Table 6:** Prevalence of moderate-or-severe and severe food insecurity according to household head, household, and geographic characteristics, Peru, 2025.

Characteristic	Moderate-or-Severe Food Insecurity				Severe Food Insecurity		
	%	±MoE	95% CI		%	±MoE	95% CI
Overall	30.9	0.7	30.2–31.6		4.0	0.3	3.7–4.3
Sex of household head							
Male	28.3	0.8	27.5–29.1		3.4	0.3	3.1–3.7
Female	35.1	1.1	34.0–36.2		5.1	0.5	4.6–5.6
Age of household head (years)							
15–29	28.9	2.6	26.3–31.5		3.7	1.1	2.6–4.8
30–59	30.4	0.8	29.6–31.2		4.0	0.3	3.7–4.3
60–74	31.8	1.3	30.5–33.1		4.1	0.5	3.6–4.6
≥75	32.2	1.8	30.4–34.0		4.6	0.8	3.8–5.4
Educational attainment of household head							
No formal education or preschool	45.6	2.4	43.2–48.0		6.6	1.2	5.4–7.8
Primary education	38.4	1.1	37.3–39.5		4.8	0.5	4.3–5.3
Secondary education	31.5	1.0	30.5–32.5		4.3	0.4	3.9–4.7
Higher education	17.9	1.1	16.8–19.0		2.3	0.4	1.9–2.7
Functional limitation of household head							
No	30.0	0.7	29.3–30.7		3.9	0.3	3.6–4.2
Yes	41.4	2.2	39.2–43.6		5.8	0.9	4.9–6.7
Household size (members)							
1 member	33.9	1.5	32.4–35.4		5.4	0.7	4.7–6.1
2–5 members	29.5	0.8	28.7–30.3		3.5	0.3	3.2–3.8
≥6 members	37.1	2.0	35.1–39.1		5.8	1.0	4.8–6.8
Household unmet basic needs							
No	29.1	0.8	28.3–29.9		3.7	0.3	3.4–4.0
Yes	41.9	1.5	40.4–43.4		6.4	0.7	5.7–7.1
Household poverty status							
Extreme poor	51.9	3.2	48.7–55.1		8.5	1.7	6.8–10.2
Poor	42.7	1.6	41.1–44.3		6.0	0.8	5.2–6.8
Non-poor	27.5	0.7	26.8–28.2		3.4	0.3	3.1–3.7
Natural region							
Coast	29.1	1.1	28.0–30.2		4.6	0.5	4.1–5.1
Highlands	32.4	1.1	31.3–33.5		2.9	0.3	2.6–3.2
Jungle	34.3	1.4	32.9–35.7		5.0	0.6	4.4–5.6
Area of residence							
Rural	37.3	1.2	36.1–38.5		3.7	0.4	3.3–4.1
Urban	29.2	0.9	28.3–30.1		4.1	0.4	3.7–4.5
Geographic domain							
Metropolitan Lima	28.5	1.8	26.7–30.3		5.0	0.9	4.1–5.9
Urban Coast	29.4	1.2	28.2–30.6		4.0	0.4	3.6–4.4
Rural Coast	35.6	3.2	32.4–38.8		5.0	1.1	3.9–6.1
Urban Highlands	28.7	1.6	27.1–30.3		2.8	0.5	2.3–3.3
Rural Highlands	37.2	1.5	35.7–38.7		3.1	0.4	2.7–3.5
Urban Jungle	32.2	1.8	30.4–34.0		4.8	0.7	4.1–5.5
Rural Jungle	38.7	2.2	36.5–40.9		5.4	0.9	4.5–6.3

Estimates were weighted using household sampling weights and account for the complex survey design of the National Household Survey (ENAHO) 2025. Moderate-or-severe and severe food insecurity were estimated using the Food Insecurity Experience Scale (FIES) following the Food and Agriculture Organization (FAO) methodology. Margins of error (MoE) were obtained directly from the FAO FIES web application https://foodandagricultureorganization.shinyapps.io/fies/ (accessed on 2 June 2026) [27], and the corresponding 95% confidence intervals were calculated as the point estimate ± MoE. Household poverty status was classified as extreme poor, poor, and non-poor according to the official poverty classification used by the National Institute of Statistics and Informatics (INEI).

## Data Availability

The questionnaire, dictionary, manuals, and databases are publicly available on the INEI website https://proyectos.inei.gob.pe/microdatos/index.htm (accessed on 27 May 2026).

## Supplementary Materials

The following supporting information can be downloaded at https://www.mdpi.com/article/10.3390/nu18152528/s1, Table S1: Sample size; Table S2: Item statistics; Table S3: Raw Score Statistics; Table S4: Rasch reliability; Table S5: Residual correlation; Table S6: Prevalence of food insecurity and margins of error (using household weights); Table S7: Probability of food insecurity by raw score; Table S8: Adjusted thresholds of food insecurity on the latent trait; Figure S1: Distributions of raw score severity; Figure S2: Scree plot of principal component analysis on residuals; Figure S3: Equating plot (Equating approach used: Maximum Discrepancy); Figure S4: Decomposition of prevalence of food insecurity by area of residence; Figure S5: Decomposition of prevalence of food insecurity by natural region; Figure S6: Decomposition of prevalence of food insecurity by department of residence; Figure S7: Decomposition of prevalence of food insecurity by sex of household head; Figure S8: Decomposition of prevalence of food insecurity by educational attainment of household head; Figure S9: Decomposition of prevalence of food insecurity by geographic domain; Figure S10: Decomposition of prevalence of food insecurity by age of household head; Figure S11: Decomposition of prevalence of food insecurity by household size; Figure S12: Decomposition of prevalence of food insecurity by household poverty status; Figure S13: Decomposition of prevalence of food insecurity by household unmet basic needs; Figure S14: Decomposition of prevalence of food insecurity by permanent functional limitation of household head; Figure S15: Decomposition of prevalence of food insecurity by raw score; Figure S16: DIF Plots by area of residence; Figure S17: DIF Plots by natural region; Figure S18: DIF Plots by department of residence; Figure S19: DIF Plots by sex of household head; Figure S20: DIF Plots by educational attainment of household head; Figure S21: DIF Plots by geographic domain; Figure S22: DIF Plots by age of household head; Figure S23: DIF Plots by household poverty status; Figure S24: DIF Plots by household size; Figure S25: DIF Plots by household unmet basic needs; Figure S26: DIF Plots by permanent functional limitation of household head.

## Abbreviations

The following abbreviations are used in this manuscript:

FIES	Food Insecurity Experience Scale
FAO	Food and Agriculture Organization
ENAHO	National Household Survey (Encuesta Nacional de Hogares)
INEI	National Institute of Statistics and Informatics
SDG	Sustainable Development Goal
MoE	Margin of Error
SE	Standard Error
CI	Confidence Interval
