# Supplementary material for: Food Insecurity in Peru: Measurement Properties of the FIES and Prevalence Estimates from the 2025 Household Survey
Source: Nutrients. 2026 Aug 4;18(15):2528. doi: 10.3390/nu18152528 (PMC13467690; doi:10.3390/nu18152528)
Supplement: Supplementary file 1 [file nutrients-18-02528-s001.zip › nutrients-4401592-supplementary.pdf]

nutrients MDPI

Supplementary Materials

Introduction

The following supplementary tables and figures were generated using the FIES web application developed by the Food and Agriculture Organization (FAO), available at https://foodandagricultureorganization.shinyapps.io/fies/ (accessed on 2 June 2026).

- Name of Survey: ENAHO 2025
- Name of Country: Peru
- FIES Items included in the survey: Worried, Healthy, Fewfood, Skipped, Ateless, Runout, Hungry, Whlday

nutrients MDPI

Supplementary Table S1. Sample size

	No.
Total No.	32041
No. complete and with non-extreme raw scores	17294
No. with at least one missing value	0

 https://doi.org/10.3390/nu18152528

nutrients MDPI

Supplementary Table S2. Item statistics

Item	Severity	Standard Error (SE)	Infit	Outfit	No. of YES among complete responses	No. missing among valid responses
WORRIED	-3.042	0.024	1.211	3.369	14824	0
HEALTHY	-2.136	0.022	0.792	1.632	12500	0
FEW-FOOD	-1.826	0.021	0.858	1.283	11598	0
SKIPPED	0.706	0.024	0.913	0.972	4552	0
ATELESS	-0.680	0.021	0.931	1.026	7846	0
RUNOUT	1.613	0.028	0.945	1.360	3042	0
HUNGRY	1.742	0.028	0.866	1.210	2750	0
WHLDAY	3.785	0.047	1.037	3.650	507	0

- Infit statistics are particularly useful to identify items that did not perform well in a given population. A low infit (below 0.7) may be somewhat redundant with other items. This usually does not substantially worsen the measure of the latent trait, but means that the item does not add important information to the measure. Particular care should be taken in the formulation of these items in future surveys to more clearly differentiate them. An adequate fit (between 0.7 and 1.3) is obtained if items are associated to the latent trait and discriminate equally well among respondents. A high infit (above 1.3) is obtained for items with unexpected response patterns. An item with high infit is considered to be performing poorly, and should be examined to decide whether to drop it from the scale for the current analysis. This also suggests that work is needed to improve this item for future surveys.
- Outfit statistics have a similar interpretation to the infit statistics, but are sensitive to the presence of even few cases with highly unexpected response patterns, and are therefore useful to flag the presence of outliers. An outfit greater than 2 is considered high. If this is due to a few highly unusual respondent response patterns (less than 0.25% of cases, e.g. 1 case out of a sample of 400), it can be ignored or the decision may be made to drop the unusual cases and refit the model, checking that the item outfit for the remaining cases is acceptable. To the contrary, if this is due to a more pervasive set of somewhat unusual patterns (there is a larger number of moderately to highly discrepant responses to the item) the decision may be made to drop the unusual cases and refit the model, checking that the item outfit for the remaining cases is acceptable. In both situations, the discrepant cases that have been dropped to refit the model should still be retained when calculating prevalence rates.

nutrients MDPI

Supplementary Table S3. Raw Score Statistics

Raw score	Severity	Standard Error (SE)	Weighted no.	Weighted proportion
0	-4.139	1.547	4,090,822	41.2
1	-3.280	1.212	1,052,183	10.6
2	-2.062	1.021	862,402	8.7
3	-1.032	1.000	1,252,555	12.6
4	-0.044	1.006	882,263	8.9
5	0.960	1.005	643,085	6.5
6	2.028	1.068	398,356	4.0
7	3.428	1.314	384,984	3.9
8	4.447	1.547	350,614	3.5

Supplementary Figure S1. Distributions of raw score severity

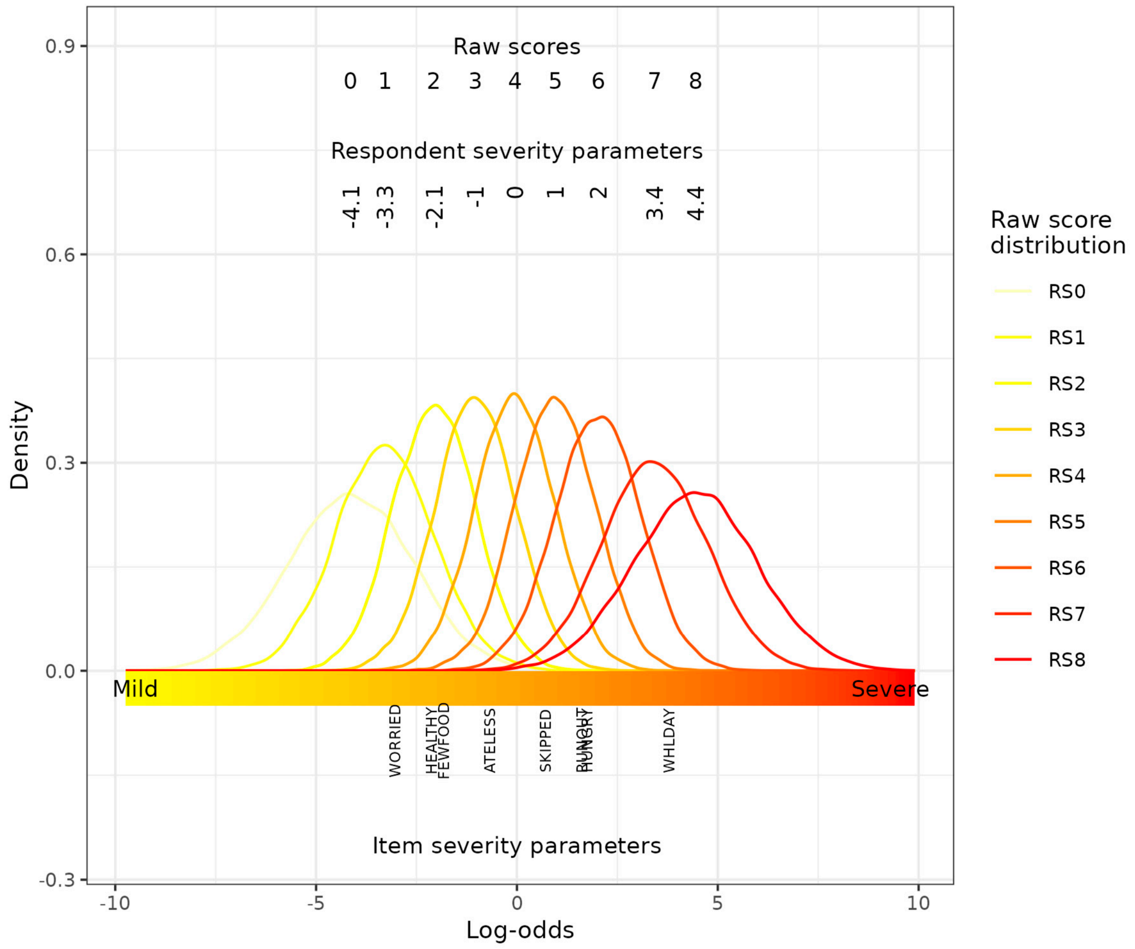

nutrients MDPI

Supplementary Table S4. Rasch reliability

Rasch reliability	0.7963321

1. Rasch reliability provides information about the discriminatory power of the overall scale, measuring the proportion of variability in the data that is explained by the Rasch model. For an 8-item FIES scale, a Rasch reliability value above 0.7 is considered acceptable. For a 7-item scale, a Rasch reliability value above 0.6 is considered acceptable.

Supplementary Table S5. Residual correlation

Item	WORRIED	HEALTHY	FEWFOOD	SKIPPED	ATELESS	RUNOUT	HUNGRY	WHLDAY
WORRIED		0.12	-0.09	-0.10	-0.17	-0.11	-0.12	-0.12
HEALTHY			0.38	-0.02	-0.07	-0.09	-0.10	-0.10
FEWFOOD				0.01	0.02	-0.08	-0.09	-0.10
SKIPPED					0.17	0.02	0.06	-0.06
ATELESS						0.06	0.06	-0.04
RUNOUT							0.24	0.06
HUNGRY								0.20
WHLDAY								

1. A residual correlation between a pair of items is considered high if it is >|0.4| (absolute term). Under the Rasch model assumptions, all correlationsResidual correlation among items should result from their common association with the latent trait (in this case food insecurity). This assumption is assessed by comparing residual correlations among items, after having eliminated the correlation that exists because the items contribute to measure the same latent trait. One possible cause of high residual correlation between one pair of items is when two items basically overlap in meaning, which may be a reflection of inaccurate translation or wording. Each item is meant to capture a different aspect of food insecurity, so having redundant questions weakens the ability to measure food insecurity accurately. One high residual correlation requires no action, however multiple high correlations suggest analyst should try eliminating one of the problematic item that is currently correlated with the others and re-run the analysis.

Supplementary Figure S2. Scree plot of principal component analysis on residuals

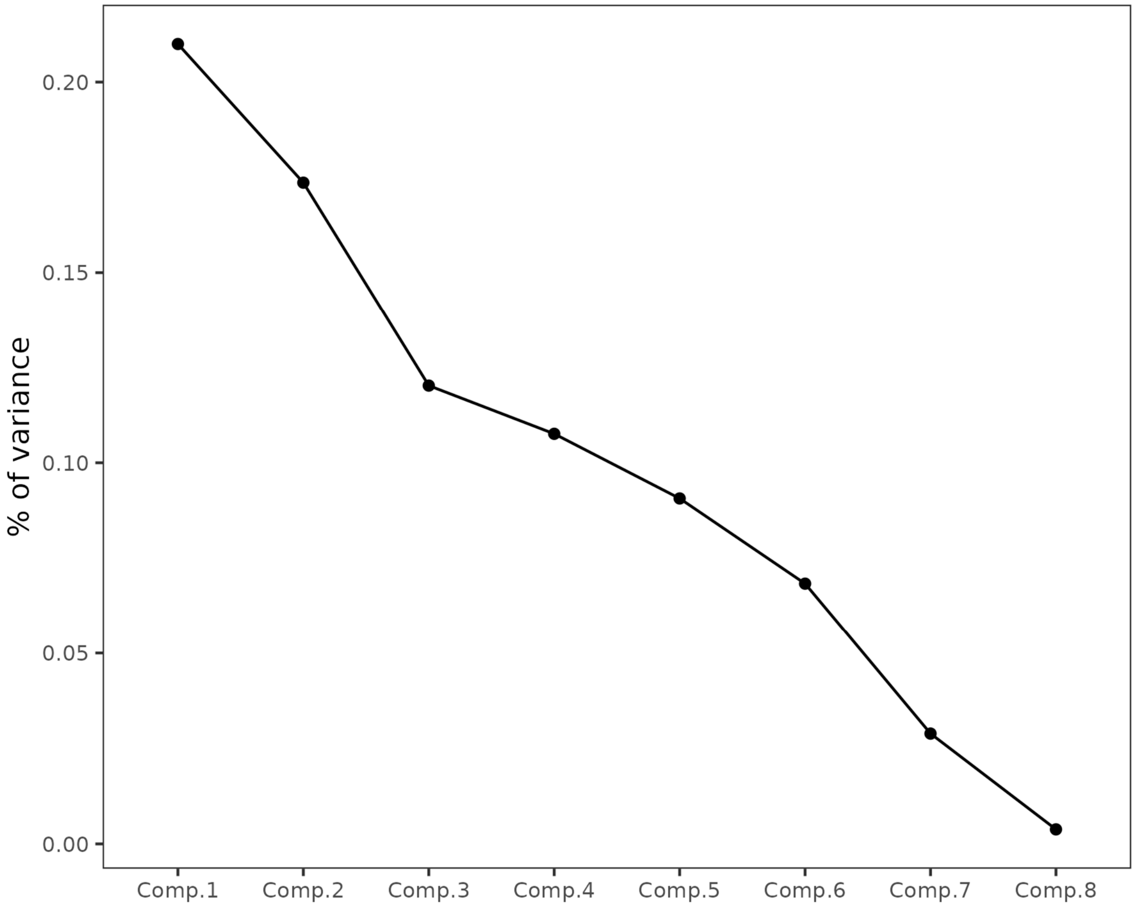

https://doi.org/10.3390/nu18152528

Supplementary Figure S3. Equating plot (Equating approach used: Maximum Discrepancy)

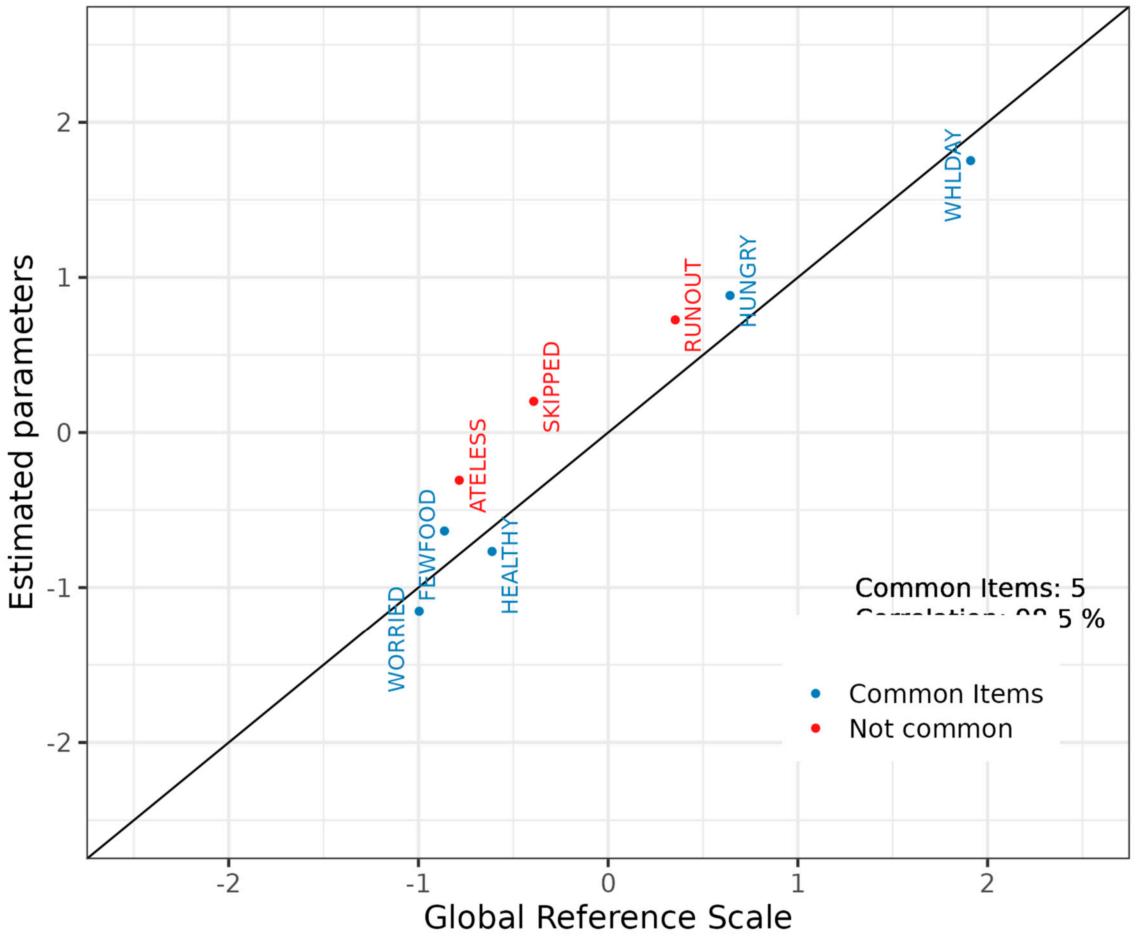

Supplementary Table S6. Prevalence of food insecurity and margins of error (using household weights)

Disaggregation	$FI_{mod+sev}$(±MoE)	FI_{sev}(±MoE)	FI_{2+}(±MoE)	FI_{3+}(±MoE)
Group: National				
Peru	30.9 (±0.7)	4.0 (±0.3)	35.3 (±0.6)	14.5 (±0.4)
Group: Area of residence				
Rural	37.3 (±1.2)	3.7 (±0.4)	43.5 (±1.2)	15.2 (±0.9)
Urban	29.2 (±0.9)	4.1 (±0.4)	33.1 (±0.9)	14.3 (±0.7)
Group: Natural region				
Highlands	32.4 (±1.1)	2.9 (±0.3)	38.1 (±1.1)	12.6 (±0.8)
Coast	29.1 (±1.1)	4.6 (±0.5)	32.7 (±1.1)	15.1 (±0.9)
Jungle	34.3 (±1.4)	5.0 (±0.6)	38.7 (±1.4)	17.3 (±1.1)
Group: Department of residence				
Lima	28.6 (±1.8)	4.7 (±0.8)	32.1 (±1.8)	15.0 (±1.4)
Loreto	45.4 (±3.0)	10.7 (±1.7)	48.2 (±3.0)	31.4 (±2.9)
Cajamarca	34.4 (±2.7)	3.0 (±0.8)	40.6 (±2.8)	12.6 (±1.7)
Piura	37.9 (±2.6)	5.9 (±1.1)	41.8 (±2.7)	21.0 (±2.2)
Moquegua	13.8 (±2.2)	1.2 (±0.6)	16.2 (±2.4)	6.2 (±1.4)
Ica	20.7 (±2.1)	1.5 (±0.6)	25.3 (±2.3)	6.7 (±1.3)
Madre de Dios	30.9 (±4.6)	7.8 (±2.4)	33.4 (±4.9)	20.5 (±3.8)
La Libertad	24.9 (±2.4)	3.0 (±0.8)	28.6 (±2.5)	11.3 (±1.6)
Apurimac	20.8 (±3.8)	1.4 (±0.9)	25.6 (±4.1)	6.8 (±2.2)
Lambayeque	36.7 (±2.8)	5.4 (±1.2)	40.8 (±2.9)	19.2 (±2.3)
Huanuco	32.2 (±3.0)	3.3 (±1.1)	37.9 (±3.1)	12.0 (±2.1)
Tacna	30.8 (±3.8)	6.4 (±2.2)	34.0 (±3.9)	18.5 (±3.5)
Tumbes	27.6 (±3.4)	3.9 (±1.3)	31.8 (±3.6)	13.2 (±2.5)

https://doi.org/10.3390/nu18152528

Disaggregation	$FI_{mod+sev}$(±MoE)	FI_{sev}(±MoE)	FI_{2+}(±MoE)	FI_{3+}(±MoE)
Arequipa	23.5 (±2.7)	2.3 (±0.9)	26.8 (±2.8)	10.8 (±1.9)
Junin	29.9 (±2.4)	3.6 (±1.0)	35.3 (±2.6)	11.2 (±1.7)
Ancash	24.0 (±2.4)	1.2 (±0.5)	30.0 (±2.6)	6.2 (±1.3)
San Martin	27.6 (±2.8)	3.1 (±0.9)	31.7 (±3.0)	11.9 (±1.9)
Amazonas	29.2 (±2.6)	1.5 (±0.6)	34.3 (±2.8)	10.5 (±1.7)
Huancavelica	34.4 (±3.4)	2.7 (±0.9)	41.2 (±3.6)	11.2 (±2.0)
Ayacucho	35.9 (±3.6)	2.9 (±1.0)	42.4 (±3.7)	12.7 (±2.3)
Cusco	35.3 (±3.4)	1.6 (±0.7)	42.5 (±3.6)	11.5 (±2.1)
Ucayali	33.8 (±3.3)	6.0 (±1.6)	37.4 (±3.4)	19.8 (±2.9)
Pasco	25.7 (±3.5)	4.3 (±1.4)	29.4 (±3.6)	12.8 (±2.6)
Callao	29.4 (±3.6)	7.2 (±2.1)	32.2 (±3.7)	18.5 (±3.2)
Puno	45.2 (±3.6)	4.3 (±1.2)	50.3 (±3.6)	21.9 (±2.9)
Group: Sex				
Hombre	28.3 (±0.8)	3.4 (±0.3)	32.5 (±0.8)	12.8 (±0.6)
Mujer	35.1 (±1.1)	5.1 (±0.5)	39.8 (±1.1)	17.3 (±0.9)
Group: Educational attainment of household head				
Primary education	38.4 (±1.1)	4.8 (±0.5)	43.9 (±1.1)	17.6 (±0.9)
Secondary education	31.5 (±1.0)	4.3 (±0.4)	35.8 (±1.0)	15.1 (±0.8)
Higher education	17.9 (±1.1)	2.3 (±0.4)	20.6 (±1.1)	8.3 (±0.8)
No formal education or preschool	45.6 (±2.4)	6.6 (±1.2)	51.8 (±2.4)	22.1 (±2.1)
Group: Geographic domain				
Rural Highlands	37.2 (±1.5)	3.1 (±0.4)	43.8 (±1.5)	13.9 (±1.0)
Urban Coast	29.4 (±1.2)	4.0 (±0.4)	33.1 (±1.2)	14.7 (±0.9)
Urban Jungle	32.2 (±1.8)	4.8 (±0.7)	36.1 (±1.8)	16.7 (±1.4)
Rural Jungle	38.7 (±2.2)	5.4 (±0.9)	44.0 (±2.3)	18.6 (±1.9)

Disaggregation	$FI_{mod+sev}$(±MoE)	FI_{sev}(±MoE)	FI_{2+}(±MoE)	FI_{3+}(±MoE)
Metropolitan Lima	28.5 (±1.8)	5.0 (±0.9)	31.8 (±1.9)	15.3 (±1.5)
Urban Highlands	28.7 (±1.6)	2.8 (±0.5)	33.6 (±1.7)	11.6 (±1.1)
Rural Coast	35.6 (±3.2)	5.0 (±1.1)	40.0 (±3.2)	18.0 (±2.6)
Group: Age of household head (years)				
60 to 74	31.8 (±1.3)	4.1 (±0.5)	36.4 (±1.3)	14.7 (±1.0)
30 to 59	30.4 (±0.8)	4.0 (±0.3)	34.7 (±0.9)	14.4 (±0.6)
15 to 29	28.9 (±2.6)	3.7 (±1.1)	32.7 (±2.7)	13.8 (±2.0)
75 or more	32.2 (±1.8)	4.6 (±0.8)	36.9 (±1.9)	15.2 (±1.4)
Group: Household size (members)				
1 member	33.9 (±1.5)	5.4 (±0.7)	38.1 (±1.5)	17.5 (±1.2)
2 to members5	29.5 (±0.8)	3.5 (±0.3)	33.9 (±0.8)	13.3 (±0.6)
6 o more members	37.1 (±2.0)	5.8 (±1.0)	41.5 (±2.1)	19.5 (±1.7)
Group: Household poverty status				
Non-poor	27.5 (±0.7)	3.4 (±0.3)	31.6 (±0.8)	12.6 (±0.5)
Poor	42.7 (±1.6)	6.0 (±0.8)	48.2 (±1.7)	20.8 (±1.4)
Extreme poor	51.9 (±3.2)	8.5 (±1.7)	57.3 (±3.2)	28.6 (±2.9)
Group: Household unmet basic needs				
No	29.1 (±0.8)	3.7 (±0.3)	33.4 (±0.8)	13.3 (±0.6)
Yes	41.9 (±1.5)	6.4 (±0.7)	46.9 (±1.5)	21.8 (±1.3)
Group: Functional limitation of household head				
Yes	41.4 (±2.2)	5.8 (±0.9)	46.7 (±2.2)	20.4 (±1.8)
No	30.0 (±0.7)	3.9 (±0.3)	34.3 (±0.8)	14.0 (±0.6)

https://doi.org/10.3390/nu18152528

Supplementary Figure S4. Decomposition of prevalence of food insecurity by area of residence

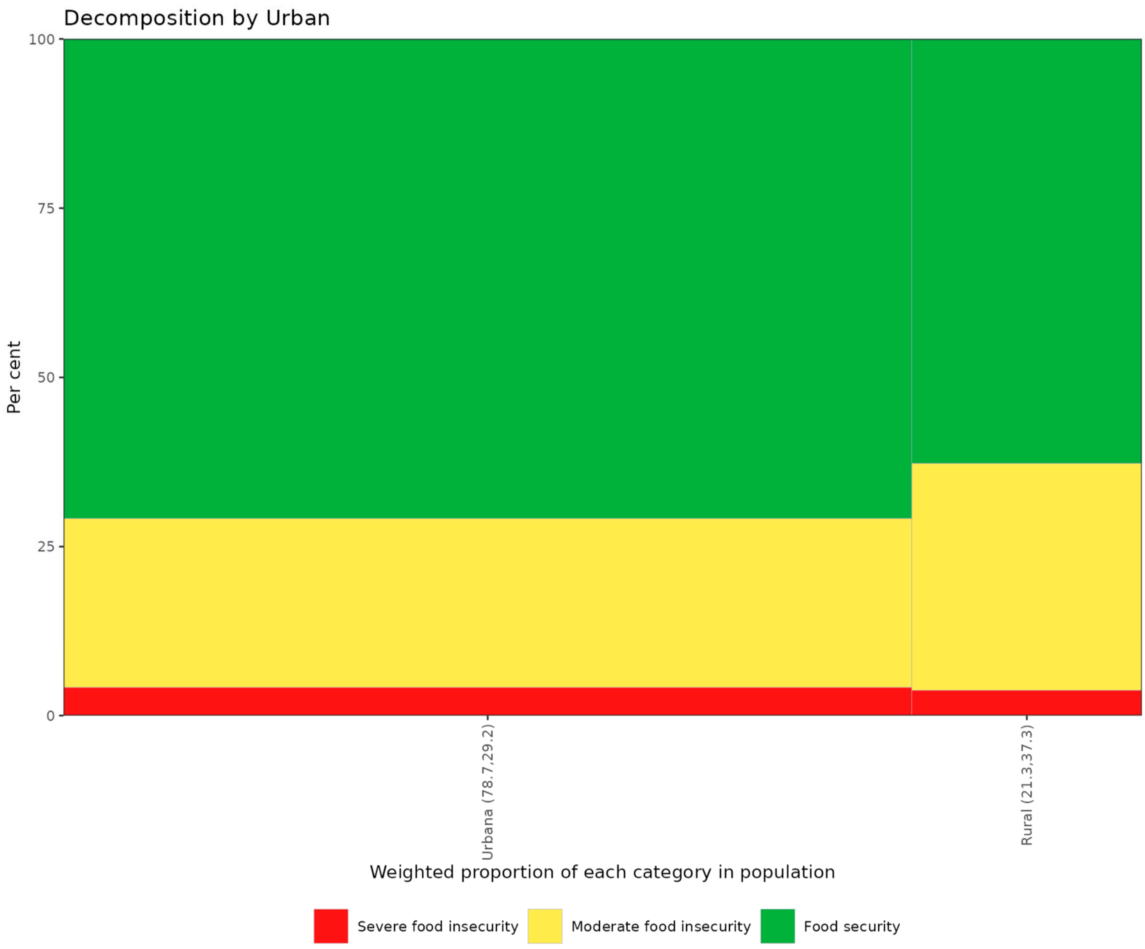

Supplementary Figure S5. Decomposition of prevalence of food insecurity by natural region

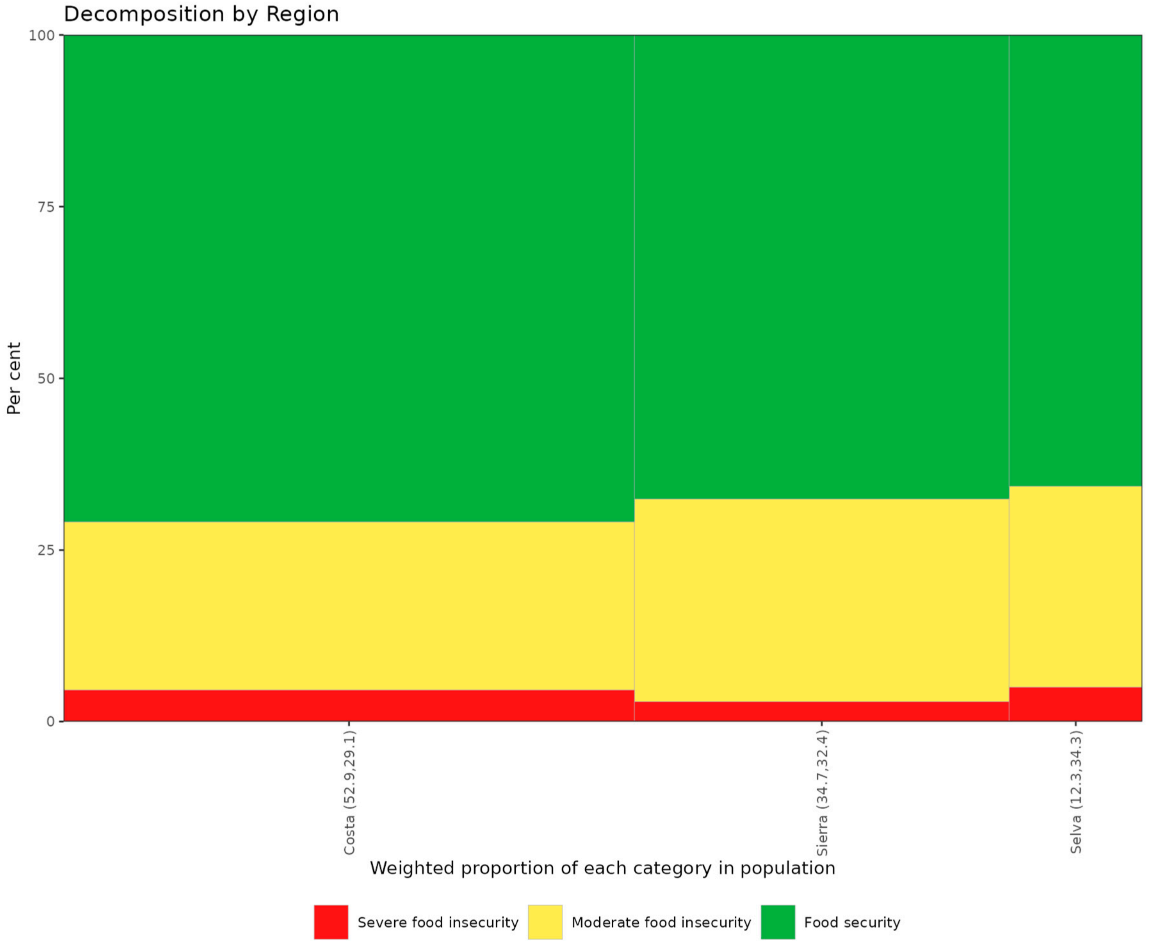

Supplementary Figure S6. Decomposition of prevalence of food insecurity by department of residence

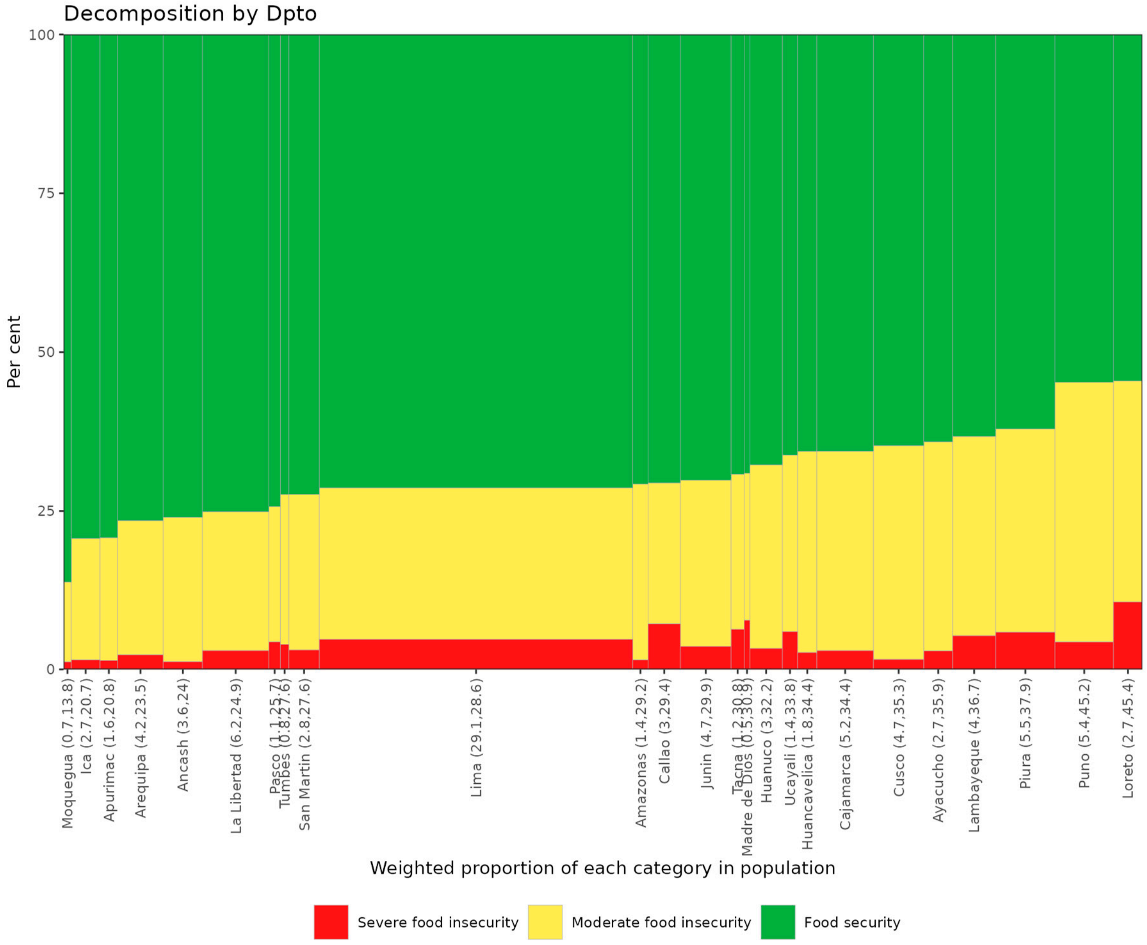

https://doi.org/10.3390/nu18152528

Supplementary Figure S7. Decomposition of prevalence of food insecurity by sex of household head

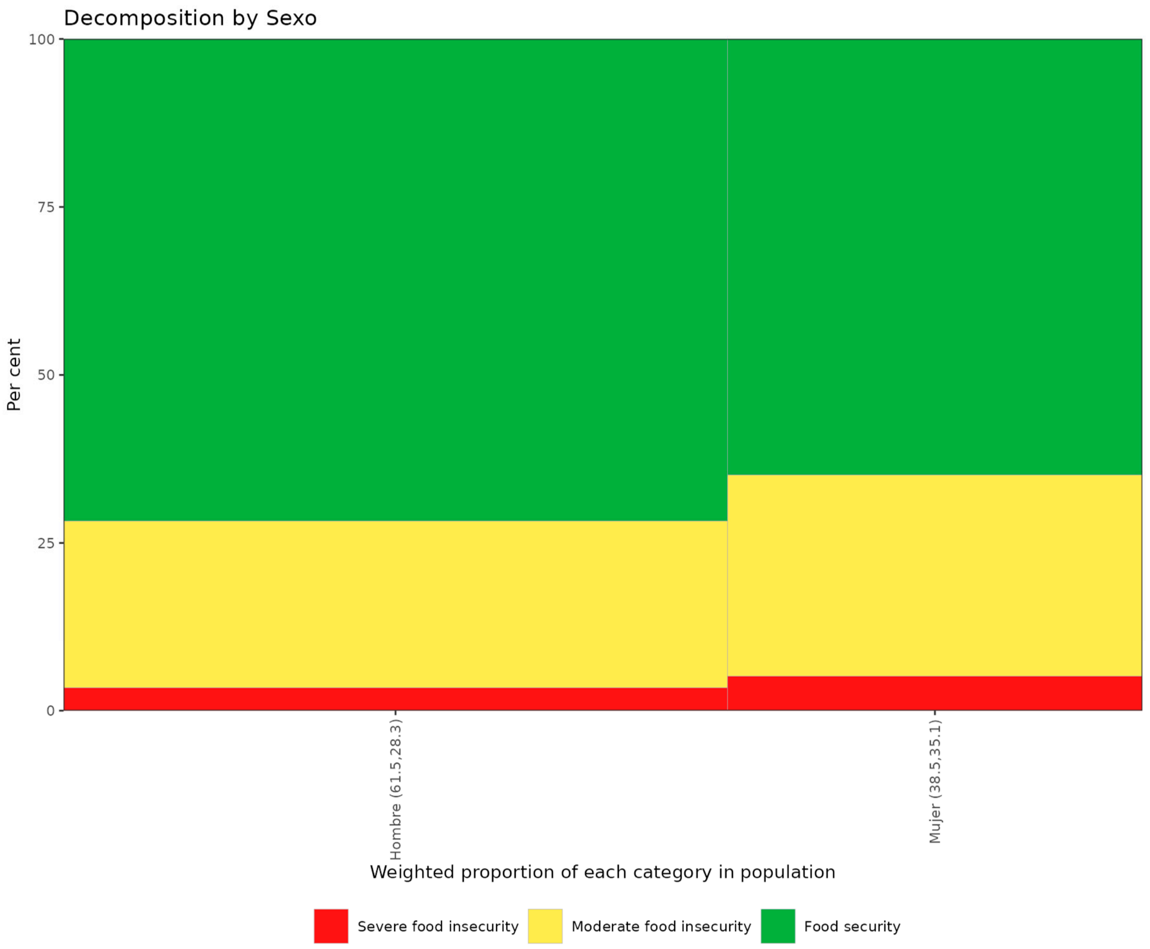

Supplementary Figure S8. Decomposition of prevalence of food insecurity by educational attainment of household head

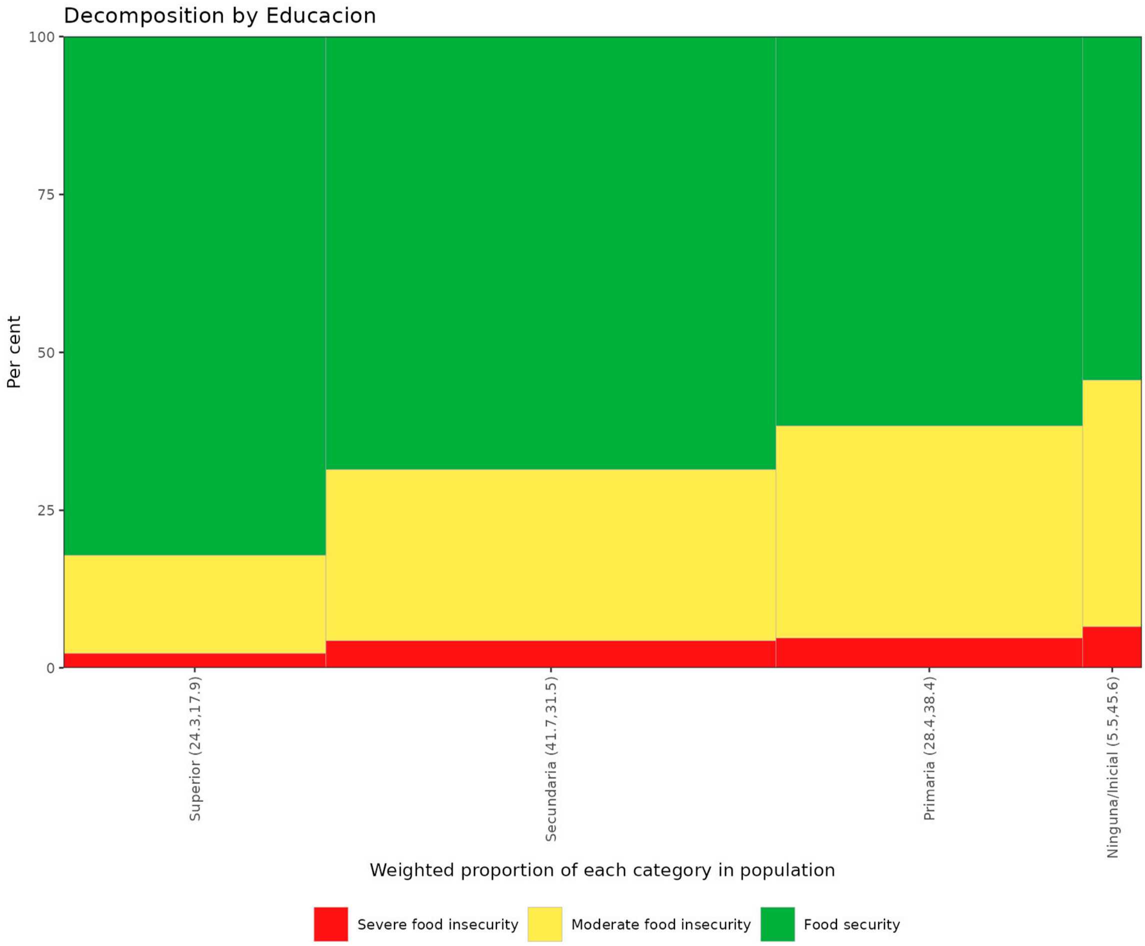

https://doi.org/10.3390/nu18152528

Supplementary Figure S9. Decomposition of prevalence of food insecurity by geographic domain

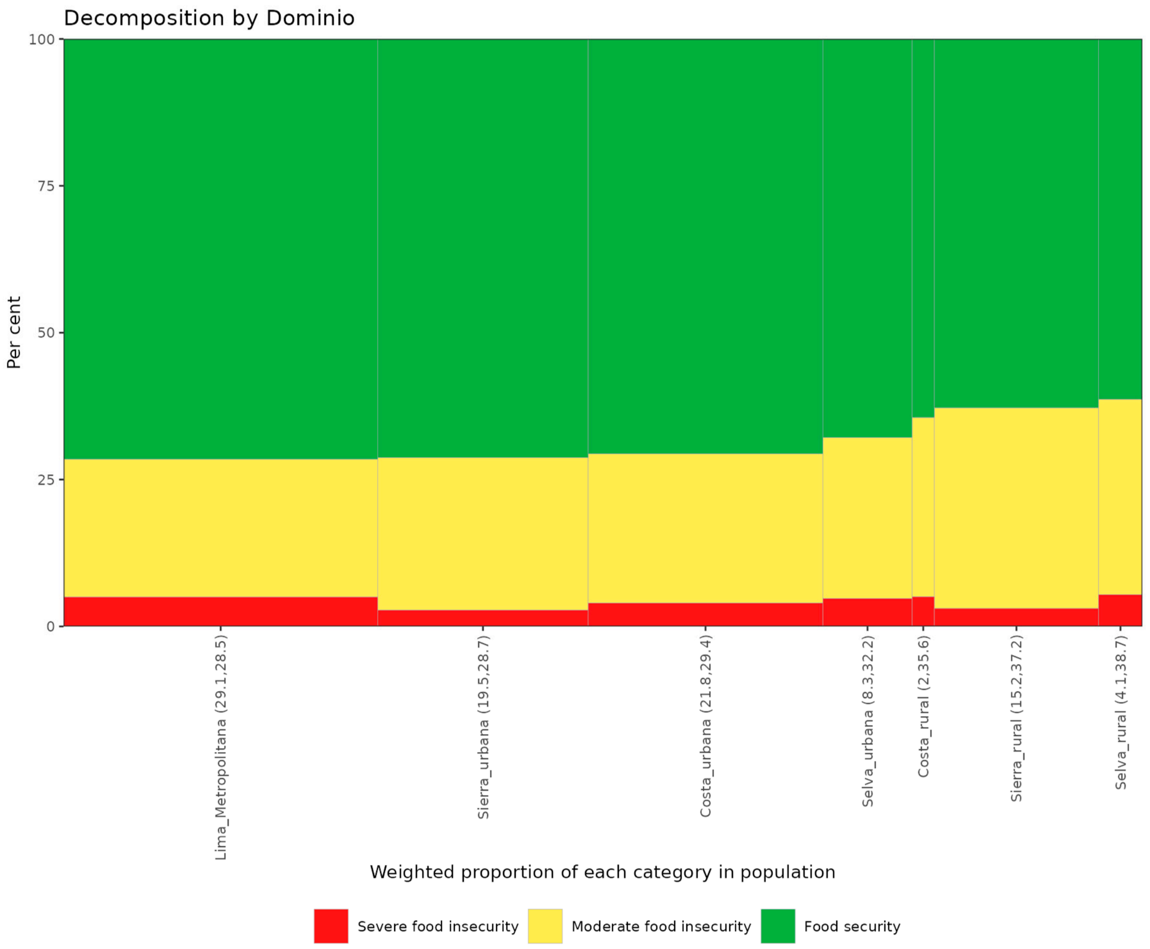

Supplementary Figure S10. Decomposition of prevalence of food insecurity by age of household head

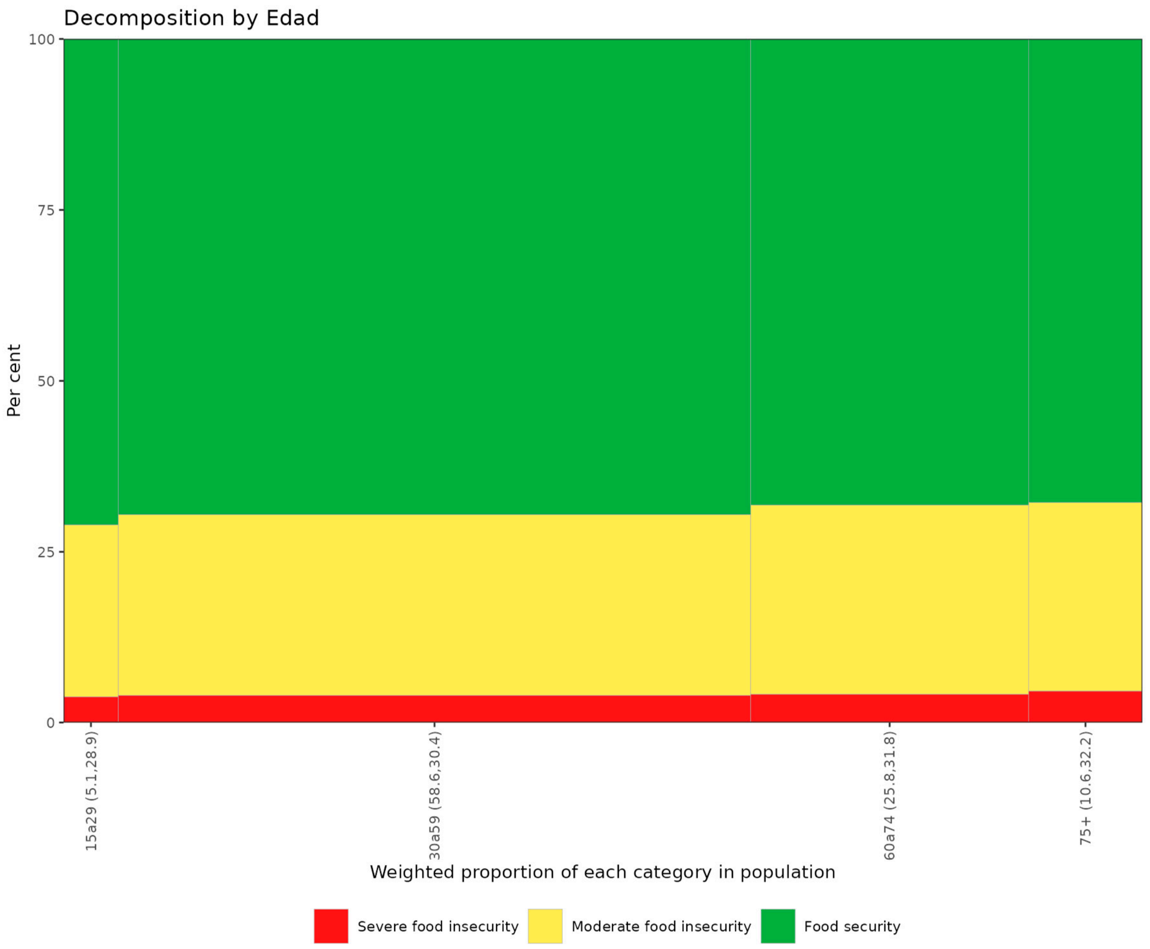

Supplementary Figure S11. Decomposition of prevalence of food insecurity by household size

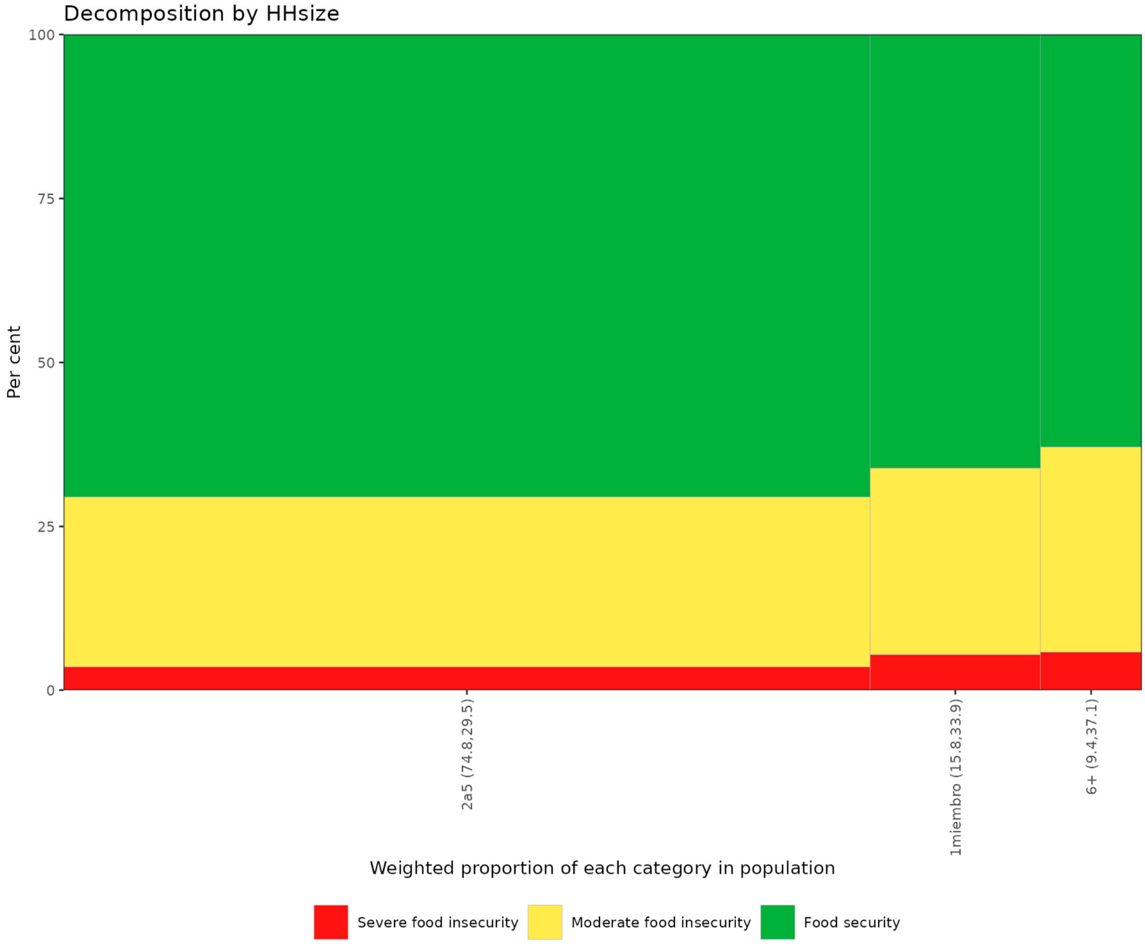

https://doi.org/10.3390/nu18152528

Supplementary Figure S12. Decomposition of prevalence of food insecurity by household poverty status

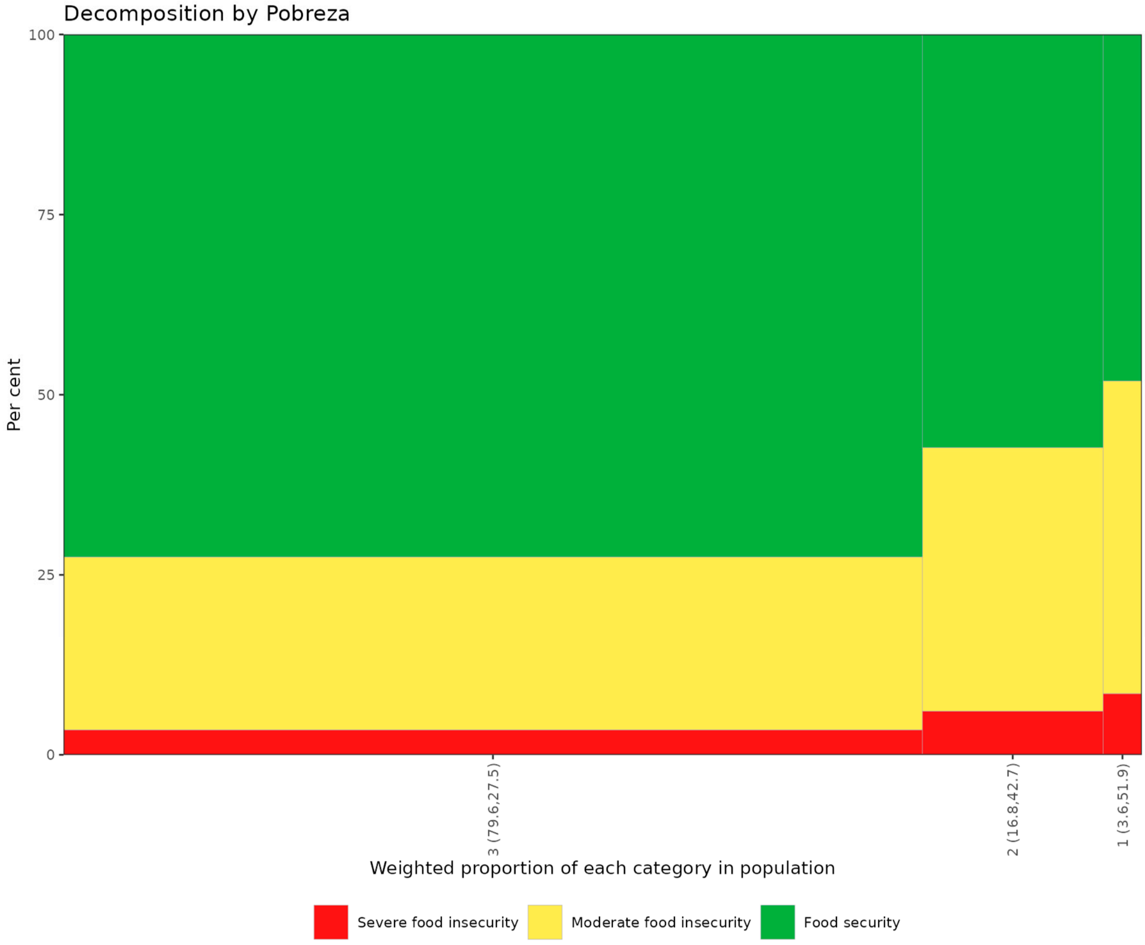

Supplementary Figure S13. Decomposition of prevalence of food insecurity by household unmet basic needs

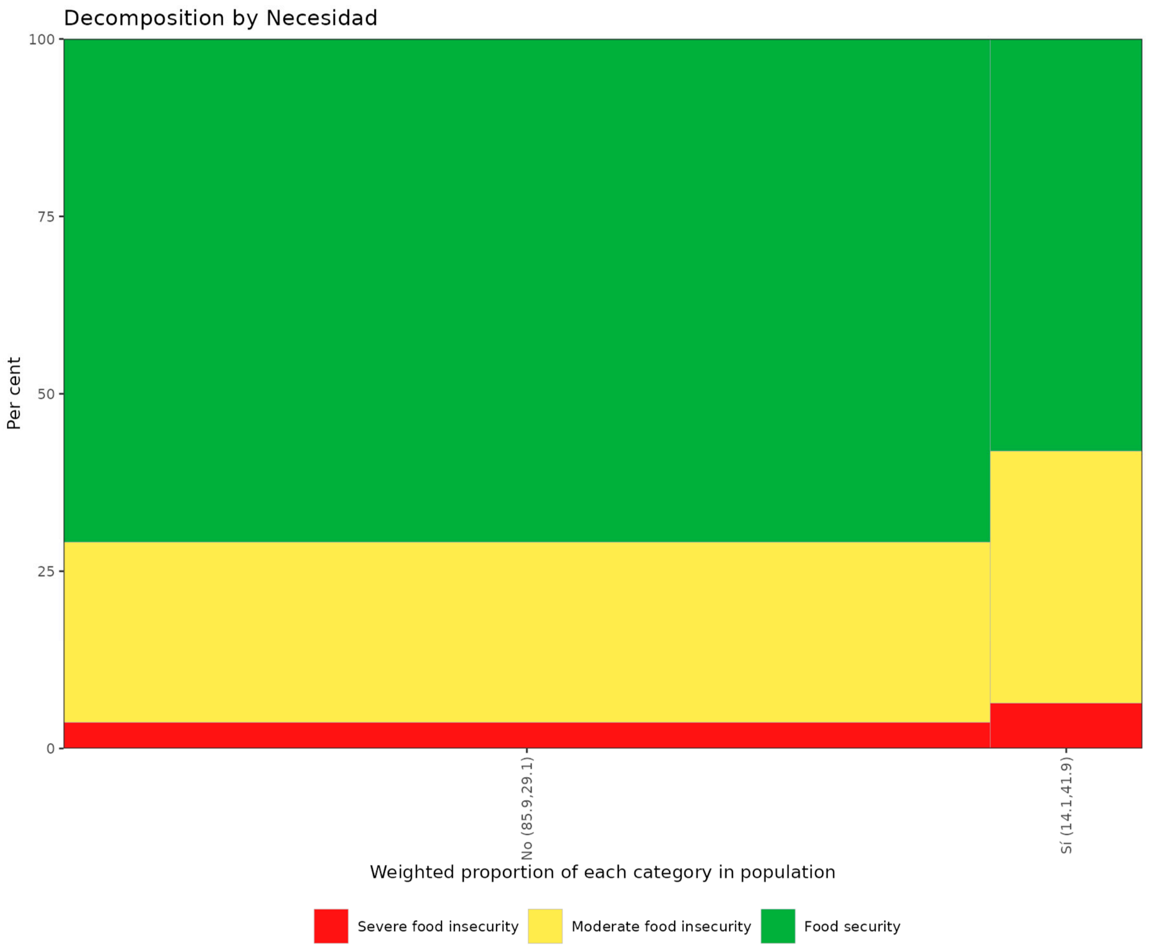

Supplementary Figure S14. Decomposition of prevalence of food insecurity by permanent functional limitation of household head

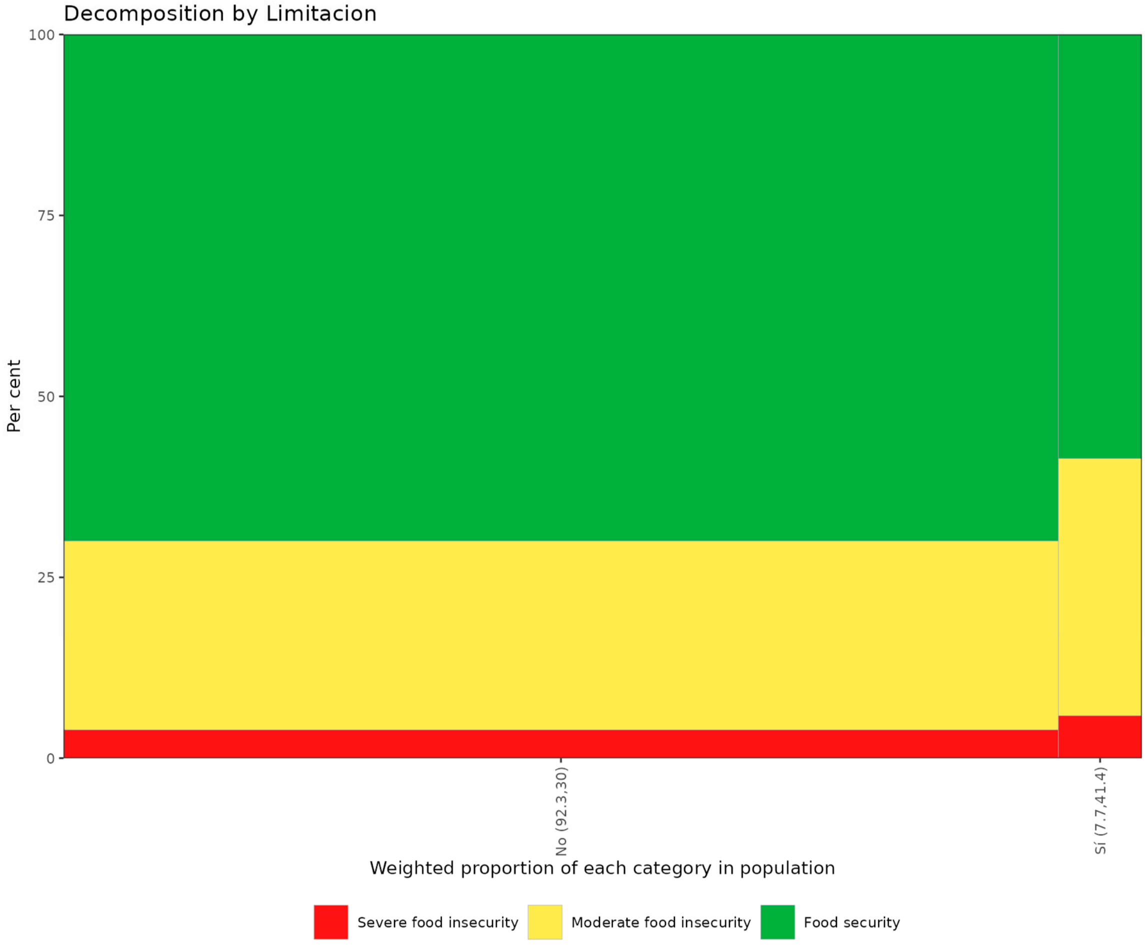

Supplementary Figure S15. Decomposition of prevalence of food insecurity by raw score

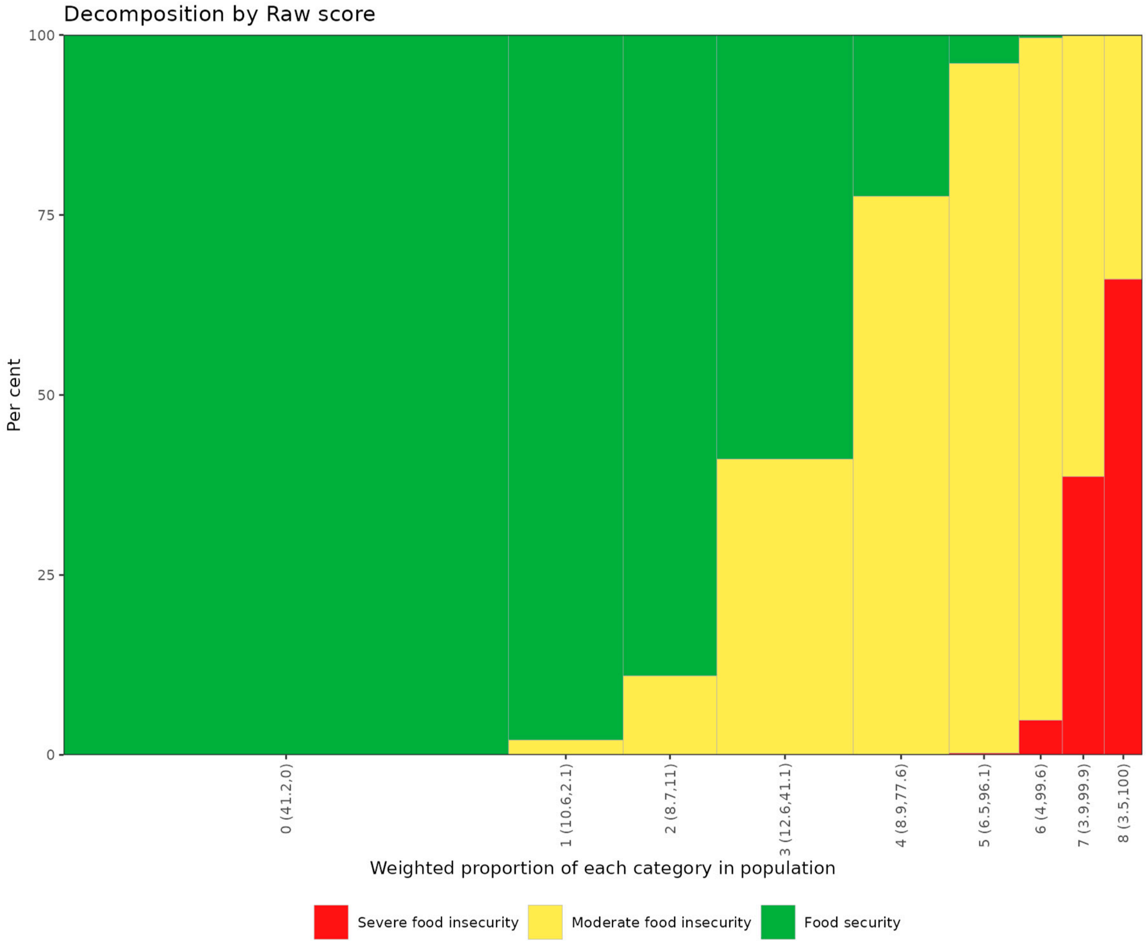

Supplementary Figure S16. DIF Plots by area of residence

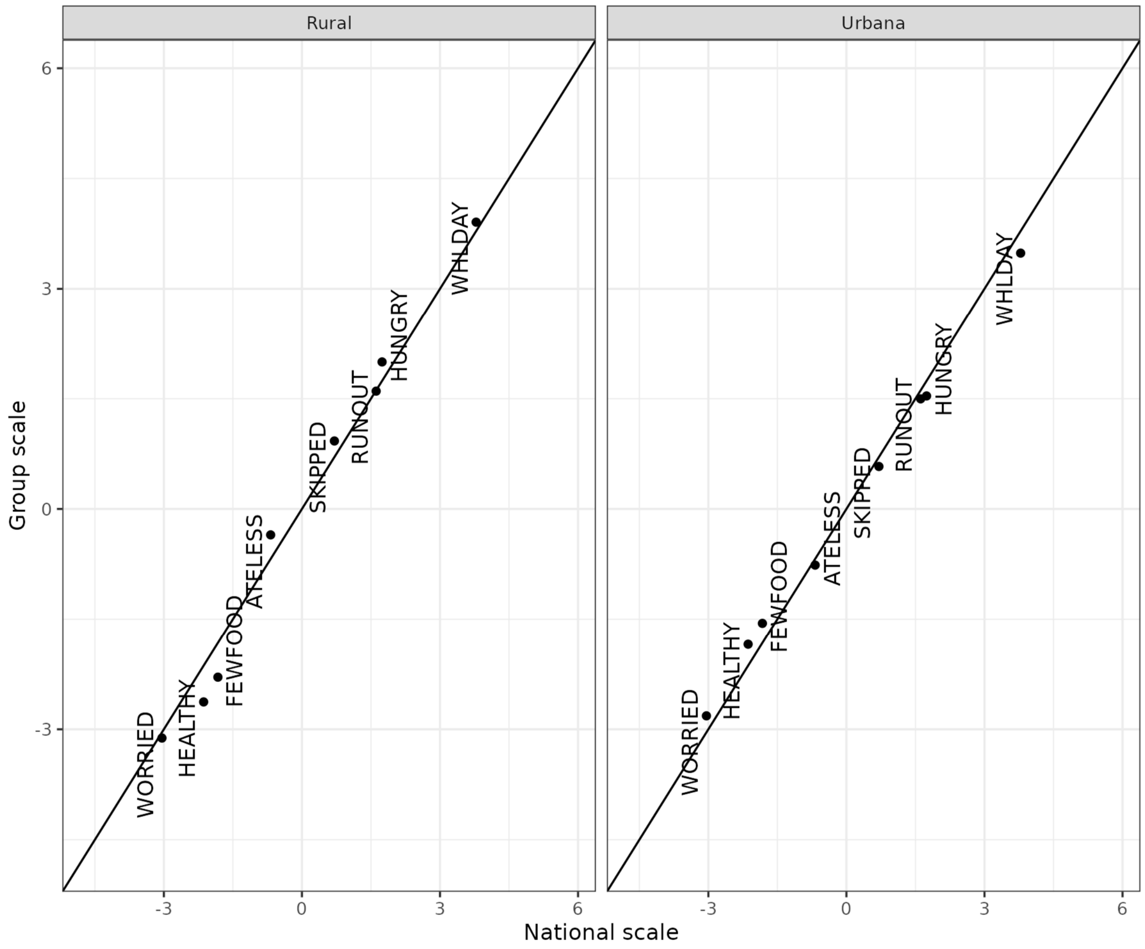

Supplementary Figure S17. DIF Plots by natural region

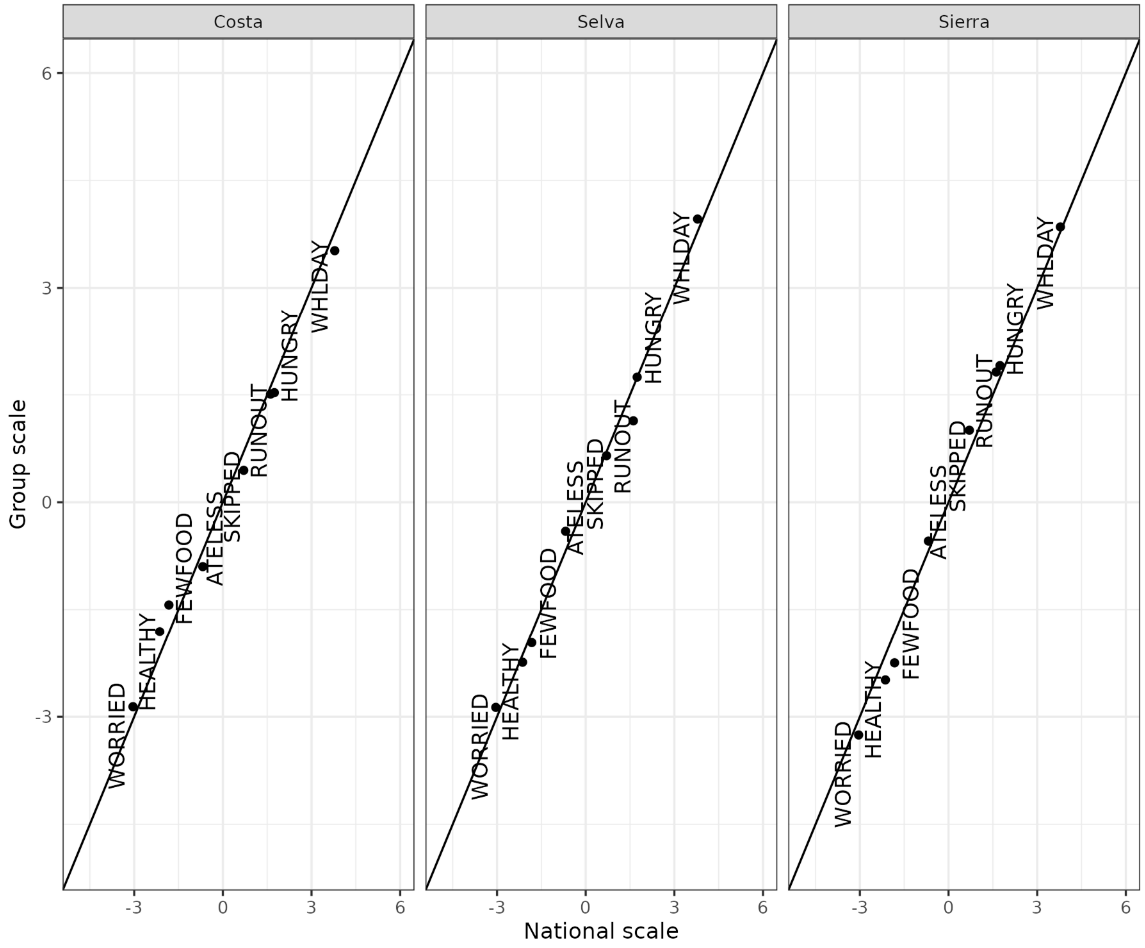

Supplementary Figure S18. DIF Plots by department of residence

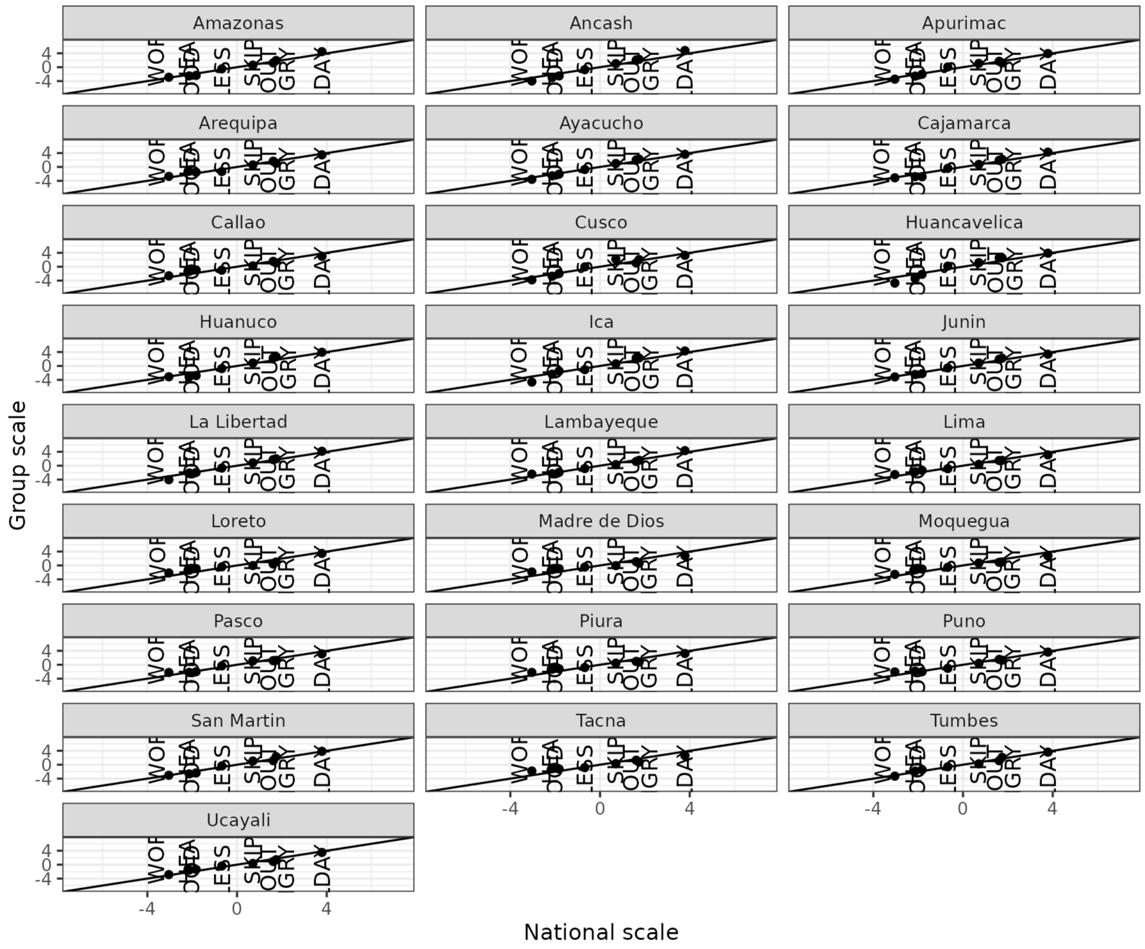

https://doi.org/10.3390/nu18152528

Supplementary Figure S19. DIF Plots by sex of household head

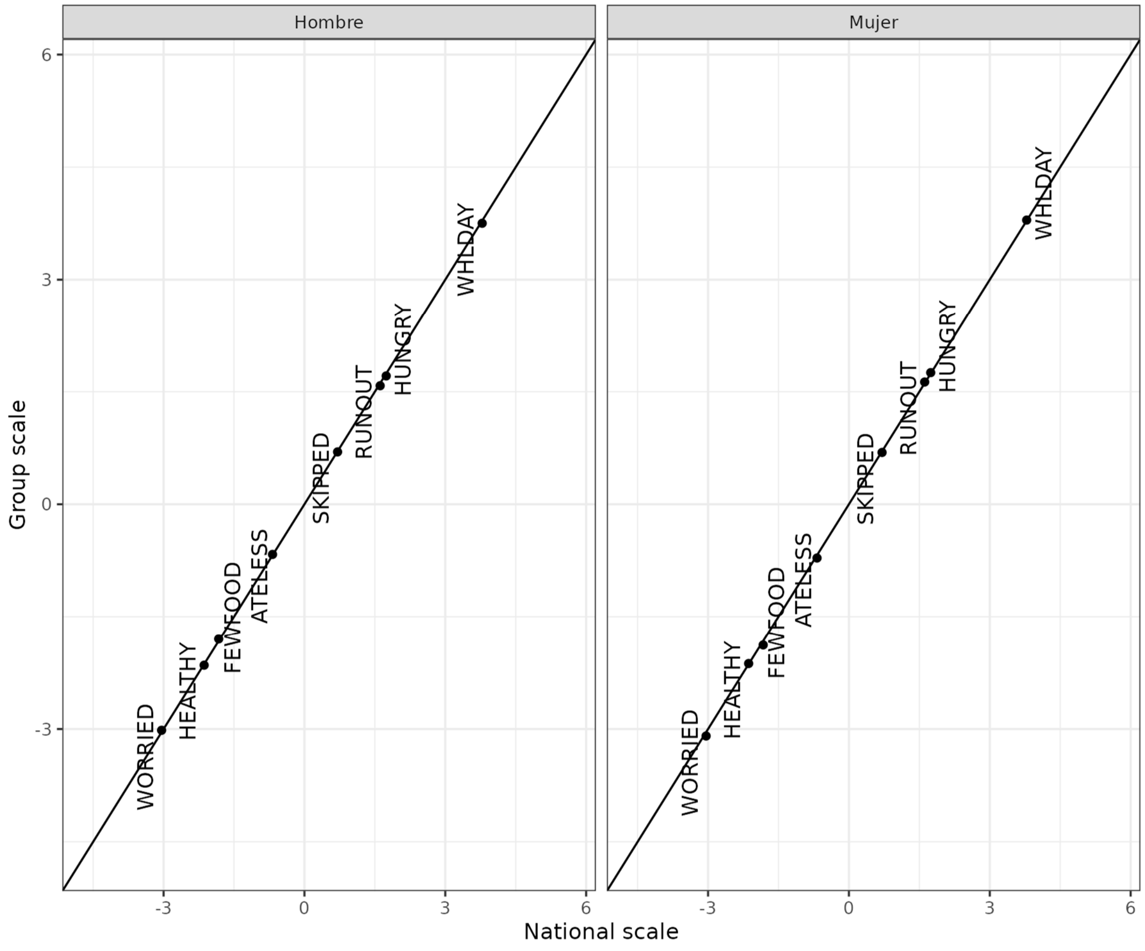

Supplementary Figure S20. DIF Plots by educational attainment of household head

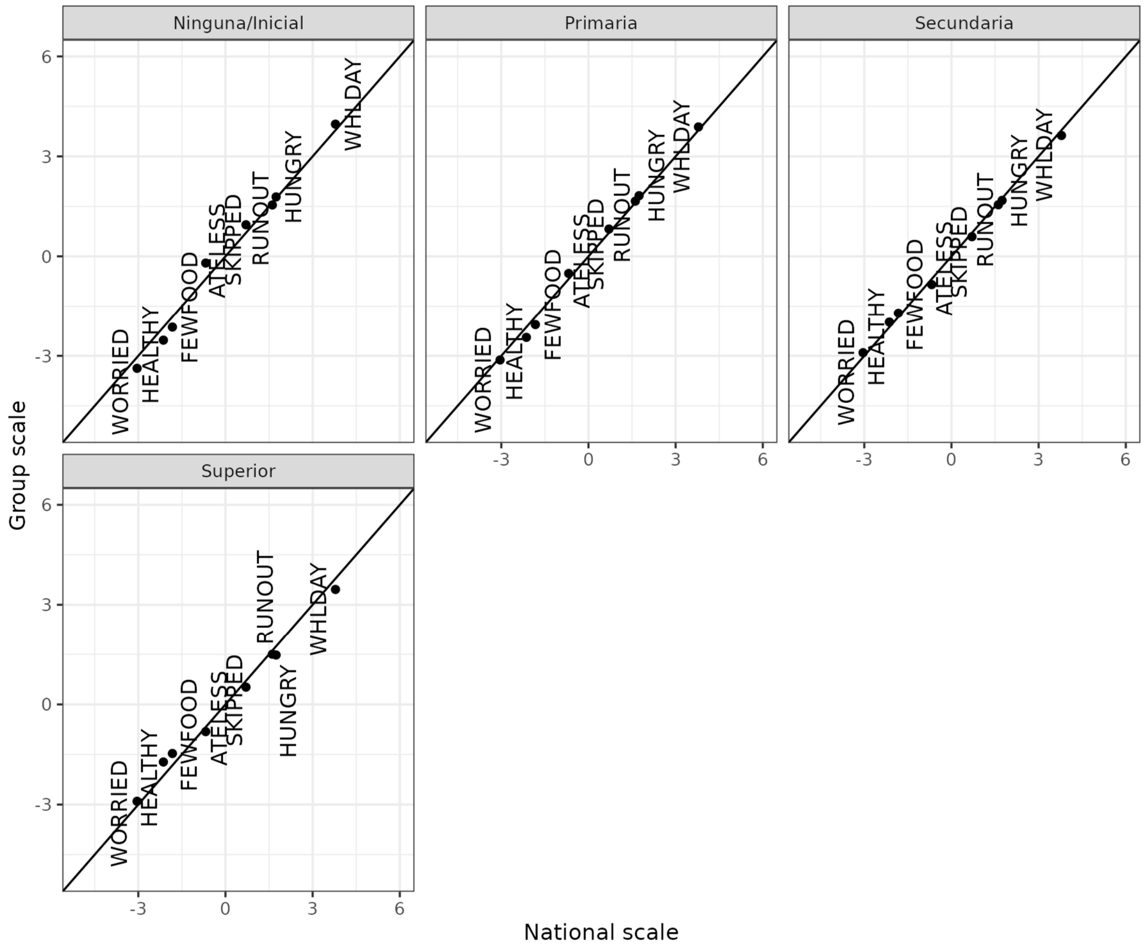

Supplementary Figure S21. DIF Plots by geographic domain

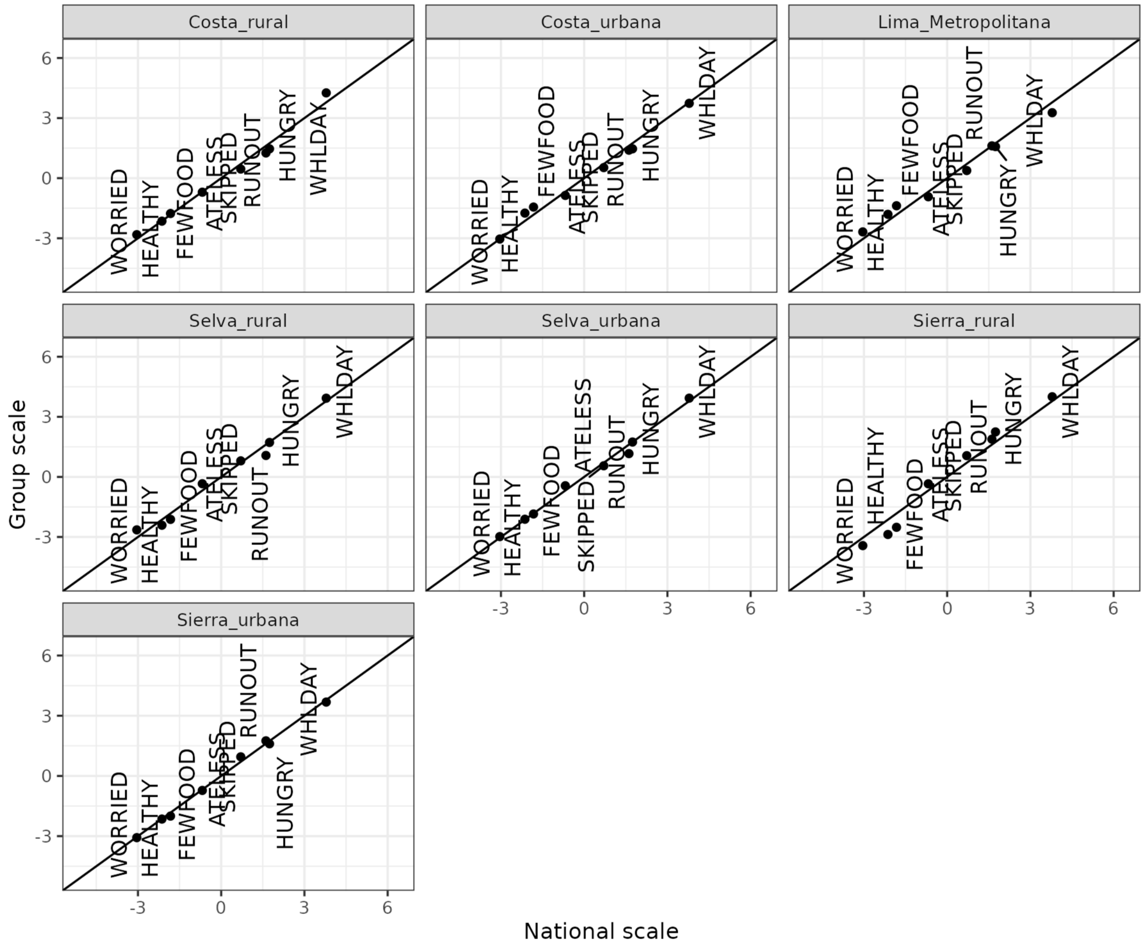

Supplementary Figure S22. DIF Plots by age of household head

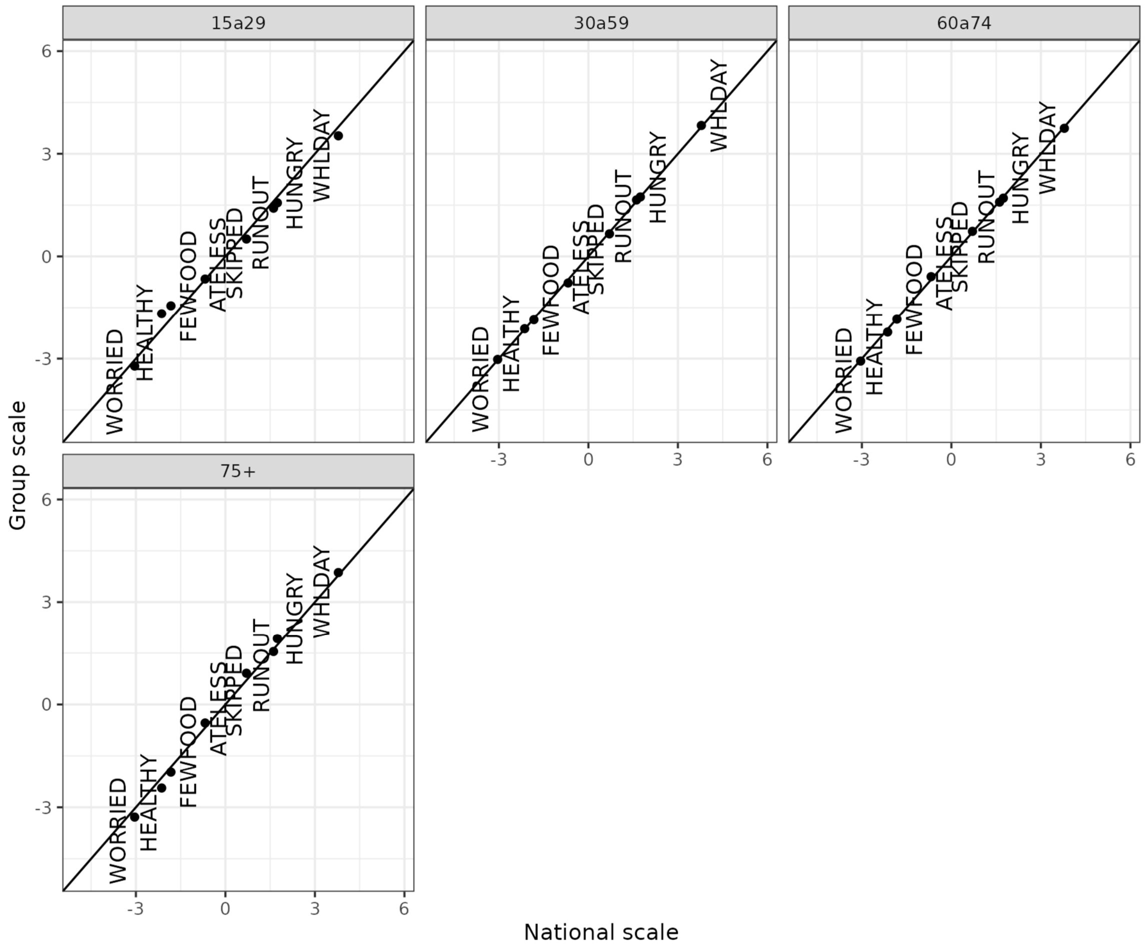

Supplementary Figure S23. DIF Plots by household poverty status

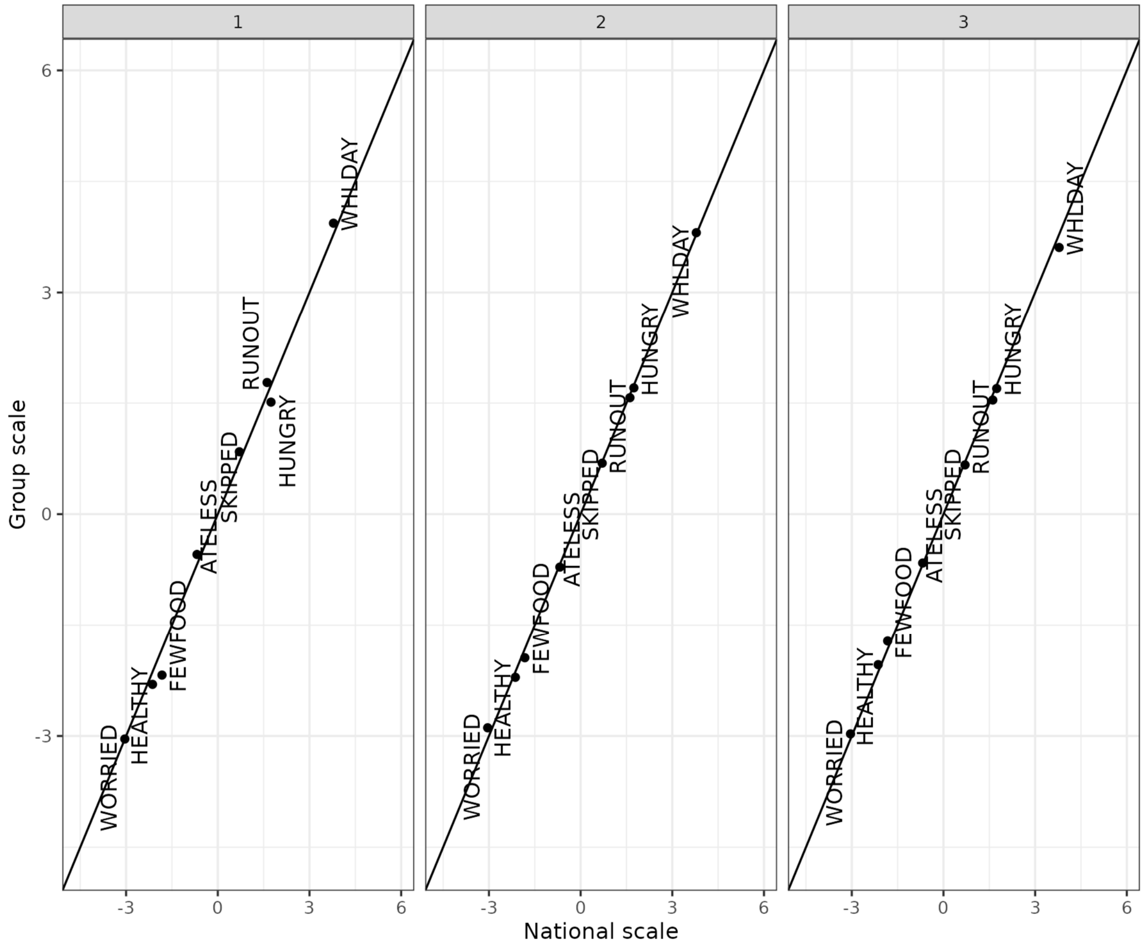

Supplementary Figure S24. DIF Plots by household size

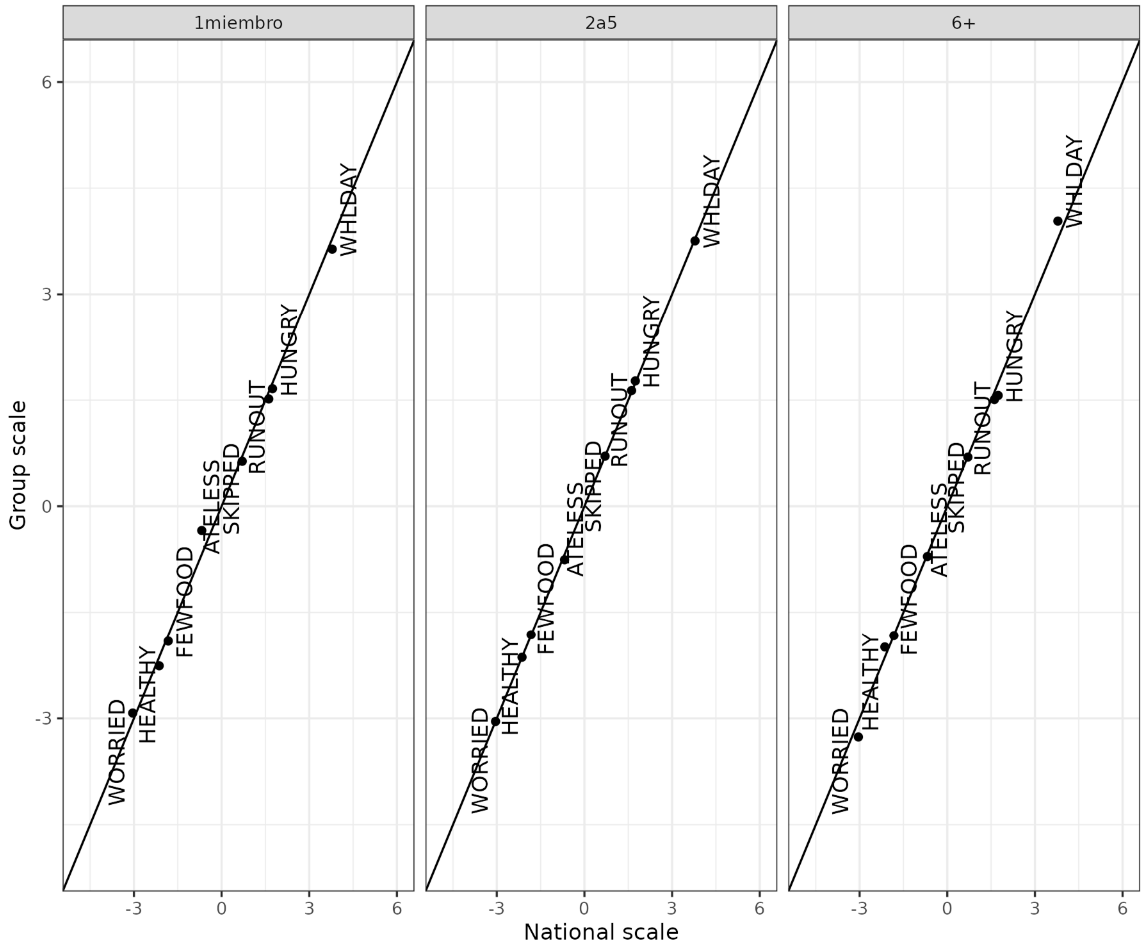

https://doi.org/10.3390/nu18152528

Supplementary Figure S25. DIF Plots by household unmet basic needs

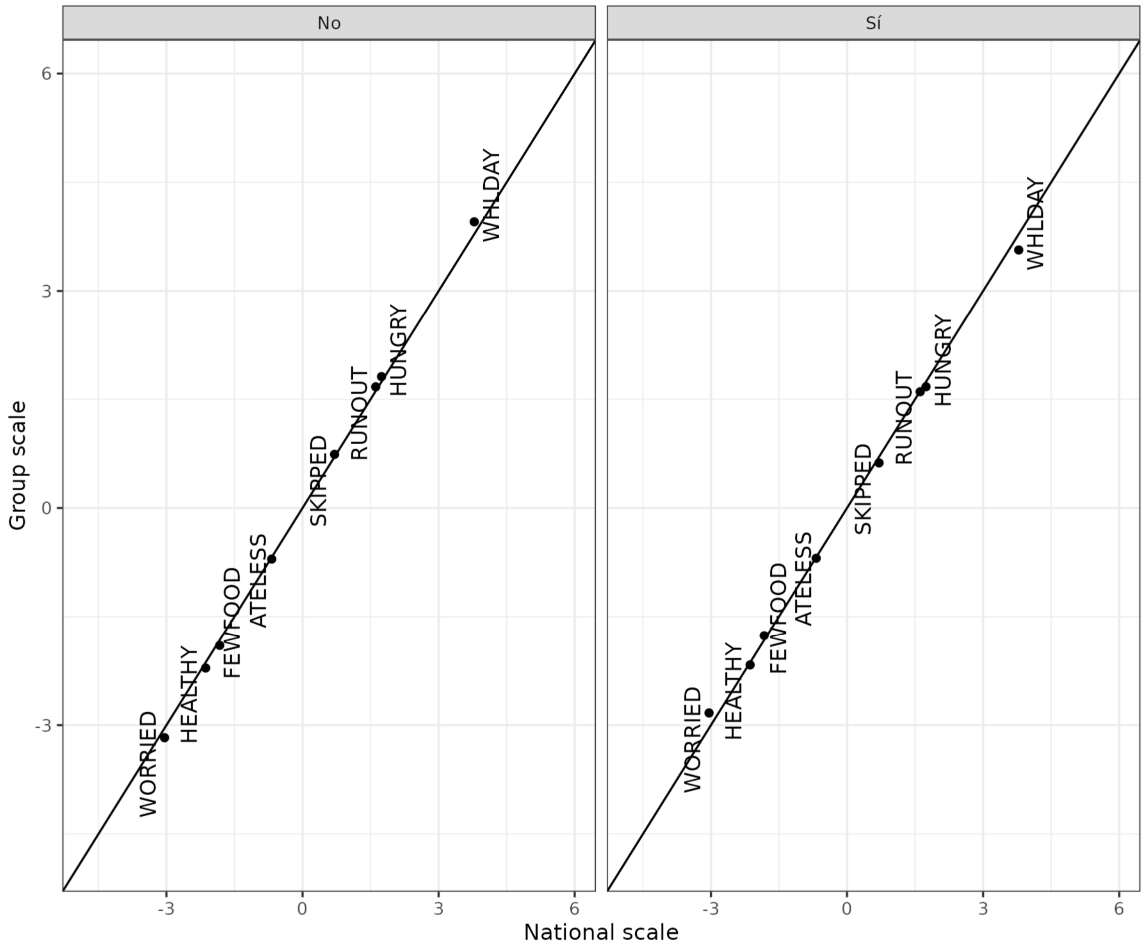

https://doi.org/10.3390/nu18152528

Supplementary Figure S26. DIF Plots by permanent functional limitation of household head

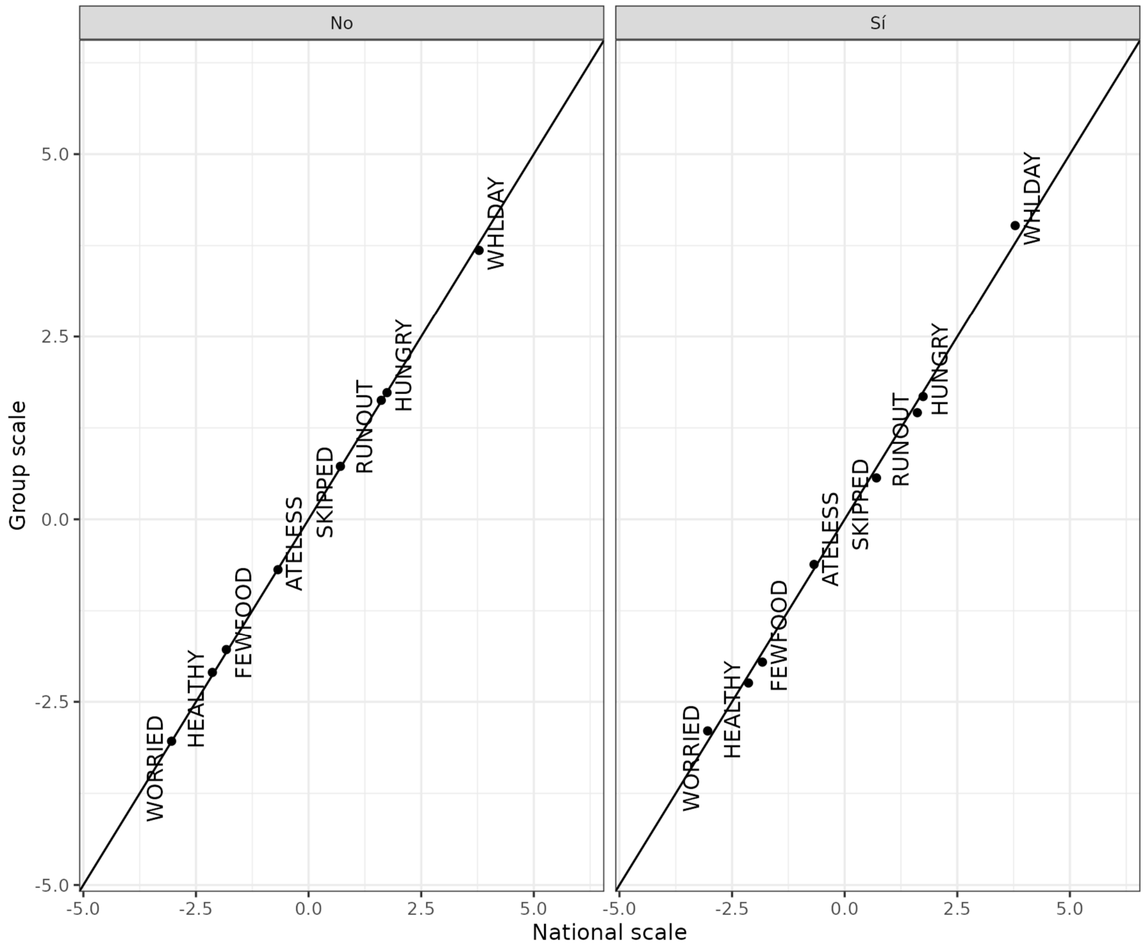

Supplementary Table S7. Probability of food insecurity by raw score

	Moderate Food Insecurity	Severe Food Insecurity	FI_3+	FI_2+
0.000	0.000	0.000	0.000	0.000
1.000	0.021	0.000	0.000	0.046
2.000	0.110	0.000	0.001	0.209
3.000	0.411	0.000	0.015	0.580
4.000	0.776	0.000	0.122	0.882
5.000	0.961	0.002	0.434	0.985
6.000	0.996	0.048	0.801	0.999
7.000	0.999	0.387	0.960	1.000
8.000	1.000	0.661	0.984	1.000

Supplementary Table S8. Adjusted thresholds of food insecurity on the latent trait

	Thresholds
Moderate	-0.81
Severe	3.81
FI_3+	1.13
FI_2+	-1.23

https://doi.org/10.3390/nu18152528
